# Load transfer in bone after partial, multi-compartmental, and total knee arthroplasty

**DOI:** 10.3389/fbioe.2024.1274496

**Published:** 2024-03-08

**Authors:** Jennifer C. Stoddart, Amy Garner, Mahmut Tuncer, Andrew A. Amis, Justin Cobb, Richard J. van Arkel

**Affiliations:** ^1^ Biomechanics Group, Department of Mechanical Engineering, Imperial College London, London, United Kingdom; ^2^ Msk Lab, Department of Surgery and Cancer, Imperial College London, London, United Kingdom; ^3^ Dunhill Medical Trust and Royal College of Surgeons of England Joint Research Fellowship, London, United Kingdom; ^4^ Nuffield Orthopaedic Centre, Oxford Universities NHS Trust, Oxford, United Kingdom; ^5^ Meshworks, Alloyed Ltd., Oxford, United Kingdom

**Keywords:** combined partial knee arthroplasty, finite element, total knee arthroplasty, unicondylar, stress shielding, strain shielding, compartmental arthroplasty

## Abstract

**Introduction:** Arthroplasty-associated bone loss remains a clinical problem: stiff metallic implants disrupt load transfer to bone and, hence, its remodeling stimulus. The aim of this research was to analyze how load transfer to bone is affected by different forms of knee arthroplasty: isolated partial knee arthroplasty (PKA), compartmental arthroplasty [combined partial knee arthroplasty (CPKA), two or more PKAs in the same knee], and total knee arthroplasty (TKA).

**Methods:** An experimentally validated subject-specific finite element model was analyzed native and with medial unicondylar, lateral unicondylar, patellofemoral, bi-unicondylar, medial bicompartmental, lateral bicompartmental, tricompartmental, and total knee arthroplasty. Three load cases were simulated for each: gait, stair ascent, and sit-to-stand. Strain shielding and overstraining were calculated from the differences between the native and implanted states.

**Results:** For gait, the TKA femoral component led to mean strain shielding (30%) more than three times higher than that of PKA (4%–7%) and CPKA (5%–8%). Overstraining was predicted in the proximal tibia (TKA 21%; PKA/CPKA 0%–6%). The variance in the distribution for TKA was an order of magnitude greater than for PKA/CPKA, indicating less physiological load transfer. Only the TKA-implanted femur was sensitive to the load case: for stair ascent and gait, almost the entire distal femur was strain-shielded, whereas during sit-to-stand, the posterior femoral condyles were overstrained.

**Discussion:** TKA requires more bone resection than PKA and CPKA. These finite element analyses suggest that a longer-term benefit for bone is probable as partial and multi-compartmental knee procedures lead to more natural load transfer compared to TKA. High-flexion activity following TKA may be protective of posterior condyle bone resorption, which may help explain why bone loss affects some patients more than others. The male and female bone models used for this research are provided open access to facilitate future research elsewhere.

## Introduction

It has been reported that, for some patients, end-stage knee osteoarthritis (OA) can result in a quality of life that is “worse than death” (Scott et al., 2019). With aging populations in many countries, the global burden of the disease is growing (Safiri et al., 2020). Arthroplasty is an efficacious, evidence-based treatment to relieve pain and restore function for people with severe OA. For at least three-quarters of people, their disease is isolated to one or two of the three knee compartments (medial, lateral, and patellofemoral) (Stoddart et al., 2021); however, total knee arthroplasty (TKA), in which all three compartments of the knee are replaced, accounts for 87% of knee arthroplasty procedures in the English and Welsh NJR (Brittain et al., 2021).

Partial knee arthroplasty (PKA), in which a single compartment of the knee is replaced and the cruciate ligaments are preserved, is a less-invasive alternative to TKA. PKA is associated with better knee function (Wiik et al., 2013; Jones et al., 2016) and lower risk of death, stroke, and myocardial infarction or blood transfusion requirement (Liddle et al., 2014). However, revision rates for cementless unicompartmental arthroplasty (UKA) are 2.7 times higher than those for cemented TKA at 15 years, while patellofemoral arthroplasty (PFA) has a revision rate in excess of five times that of TKA at 10 years (Brittain et al., 2021). In 2022, for the first time, the NJR reported on revision rates for combined (i.e., multi-compartmental) partial knee arthroplasty (CPKA). While the recorded numbers are small and should be interpreted with caution, their revision rates are more than four times higher than that for TKA at 5 years. The reasons for higher revision rates in PKA are complex and multi-factorial (Goodfellow et al., 1976); however, the progression of arthritis in an unresurfaced knee compartment remains a leading cause. CPKA allows for the preservation of a well-functioning, well-fixed PKA in these cases (Garner et al., 2021f), or an alternative primary procedure for the one-in-three patients with bicompartmental knee disease (Garner et al., 2019; Stoddart et al., 2021). CPKA is associated with favorable patient outcomes (Biazzo et al., 2018; Wada et al., 2020; Garner et al., 2021e; Garner et al., 2021f; Garner et al., 2021b), stability (Garner et al., 2021d), and function (Wang et al., 2018; Garner et al., 2021b; Garner et al., 2021) compared to TKA. However, it is not known how use of multiple PKAs in the same knee affects load transfer to bone.

Preventing arthroplasty-associated bone loss remains an unmet clinical need, with aseptic loosening being the leading cause of implant failure associated with TKA (Brittain et al., 2021). Bone remodeling is sensitive to mechanical stimulus: increased strain can lead to bone formation, bone structure optimization, and higher bone mineral density (BMD), while reduced strain leads to bone resorption and bone loss (Huiskes et al., 1987). Finite element analysis enables the quantification of the change in the strain environment and is widely used to understand how interventions and disease affect load transfer and bone remodeling (Ong et al., 2009; Dickinson, 2014; Quilez et al., 2017; Zhang et al., 2020; Anijs et al., 2022). CPKA procedures could, in theory, lead to favorable load transfer to bone, as articulating surfaces, ligaments, and bone stock are preserved compared to TKA. However, it is also possible that the regions of bone between implants could lead to unfavorable loading conditions that could, perhaps, accelerate bone loss (through strain shielding) or risk of fracture (through overstraining) (Stoddart et al., 2022).

The aim of this research was to analyze how the different knee arthroplasty options (PKA, CPKA, and TKA) affect load transfer to bone using the finite element method. It was hypothesized that preserving the intact joint surface in CPKA would result in more favorable load transfer to bone, indicative of reduced risk of bone loss following arthroplasty.

## Materials and methods

### Bone model preparation

An experimentally validated (Tuncer et al., 2013) subject-specific (Supplementary Table S1) finite element model (female, right-sided) of the distal femur and proximal tibia was prepared intact, with PKAs: medial (UKA-M) and lateral (UKA-L) unicondylar, PFA- CPKAs: bi-unicondylar (Bi-UKA), medial (BCA-M) and lateral (BCA-L) bicompartmental, and tricompartmental (TCA), and TKA. All implants were cemented variants from the same manufacturer (Zimmer Biomet, United States): the mobile bearing medial Oxford® and fixed lateral Oxford® (FLO) UKA implants, the Gender Solutions® PFA, and the NexGen® cruciate retaining (CR) TKA. Standard NexGen® CR implants were used and did not include the option tibial components or any combinations subject to the voluntary medical device field safety corrective action conducted by Zimmer Biomet Inc. Since the combination of implants used in the tibia was identical for UKA-M and BCA-M, UKA-L and BCA-L, and Bi-UKA and TCA, only the models for UKA-M, UKA-L, and Bi-UKA were prepared for this study.

Implants were sized and positioned using surgical planning software (Embody Orthopaedic, UK), following the senior surgical author’s clinical practice (Supplementary Table S2). Boolean operations were used to prepare the bones for the implants (including a 1 mm cement layer), and the volume of bone removed was quantified for each procedure.

Bone volume meshes were created in 3-matic (Materialise NV, Belgium) with ten-noded tetrahedral elements. The cortical bone was modeled with type 127 ten-noded tetrahedral elements in MARC (MSC Software Corporation, United States). The thin tibial cortex was modeled with type 22 one-side collapsed quadratic quadrilateral 0.2 mm thick shell elements to reduce partial volume effects arising from the bone material allocation method. Mesh convergence was assessed for the accuracy of the equivalent elastic strain predicted by each bone model under toe-off in gait loading in nine regions of interest: at the tips of each peg in the distal femur implanted with TCA and around the keels of each implant in the proximal tibia implanted with Bi-UKA. An element edge length of 2 mm resulted in peak values in each region of interest that were within 5% equivalent elastic strain of those predicted by the highest-density mesh investigated.

### Material properties

The bones were CT-scanned (Definition AS+, Siemens, Germany) with a slice thickness of 0.6 mm and cross-section voxels 0.5 × 0.5 mm. The scans were phantom-calibrated against air and water: the grey-scale values were calibrated as Hounsfield units (HU) such that water corresponds to ±4 HU and air to −1,000. Cancellous bone material properties were applied heterogeneously based on empirical measures relating HU to density and elastic moduli from quantitative CT of each specimen using Mimics (Materialise NV) (Tuncer et al., 2013). All other materials were homogeneous, isotropic, and linear elastic (Supplementary Table S3).

### Boundary conditions

**FIGURE 1:**
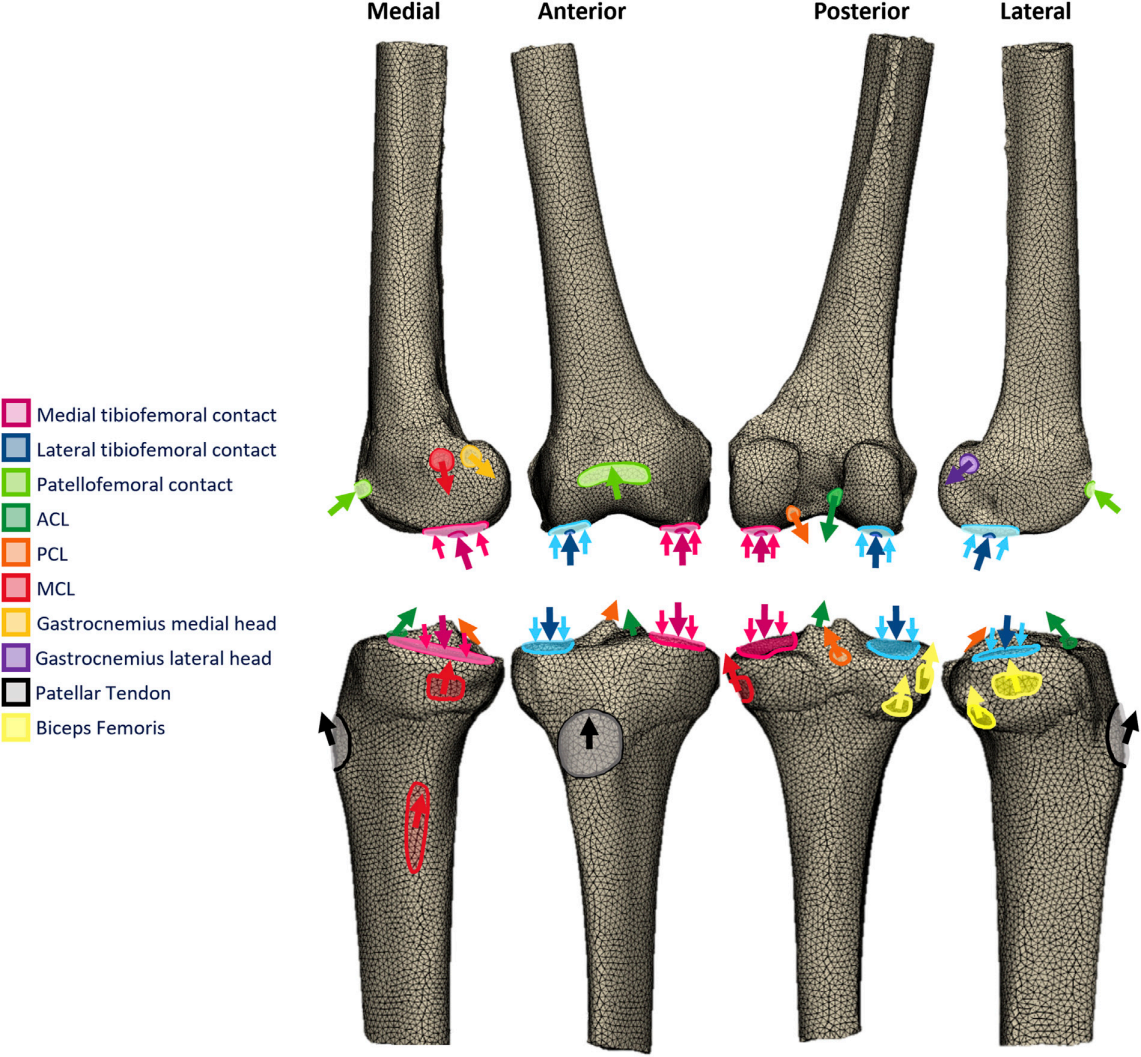
Free-body diagrams illustrating the toe-off in gait loading applied to the femoral and tibial finite element models.

The most proximal and distal surfaces of the femoral and tibial diaphysis, respectively, were fixed. Loading for three activities of daily living was modeled to capture the effects of load transfer for a range of contact locations: gait (toe-off, 15^o^ flexion, when tibial contacts the distal condyles, and when patellar contacts the proximal trochlea) (Figure 1), stair ascent (weight acceptance, 50^o^ flexion, when tibial contacts the posterior plateaux, femoral posterodistal condyles, and when patellar contacts the mid trochlea), and sit-to-stand (when leaving chair, 90^o^ flexion, when tibial contacts the posterior plateaux, femoral posterior condyles, and when patellar contacts the distal trochlea).

The tibiofemoral joint reaction force was based on measured *in vivo* telemetric data for posterior-stabilized TKA (Bergmann et al., 2014) scaled to the bodyweight of each model specimen. The mediolateral load split was calculated according to the varus–valgus moment at the point of peak contact force (Kutzner et al., 2013). Equivalent data are not available for the native knee, PKA/CPKA, or posterior-cruciate retaining TKA. The data were, thus, adapted and applied to the models as follows: 1) The loading magnitude and medial–lateral distribution were assumed to be the same for all variants. This allowed for direct comparisons where the only change was the type of implant. 2) Equal and opposite musculoskeletally predicted ACL and PCL forces were added to the tibiofemoral contact forces appropriately for each modeling condition, since neither were present in the orthoload data (i.e., ACL and PCL for the native, PKA, and CPKA states and PCL only for the TKA state). 3) In native knee compartments, condylar loads were distributed to simulate the effect of the menisci and cartilage, in accordance with a method that has previously been verified (Stoddart et al., 2022) with lab data (Munford et al., 2022). 4) Loading through implants was applied as point load to the implant, in accordance with the previous model validation (Tuncer et al., 2013).

Muscle and ligament forces associated with each load case were derived from the results of published musculoskeletal models (Rasnick et al., 2016; Hume et al., 2019). Muscle and ligament forces were applied directly to the bone models as point loads distributed over anatomically derived attachment areas, rather than as continuum bodies. Other than the presence or absence of the ACL, the load cases applied to the intact and implanted bones were identical. The directions of the muscle force vectors were determined by inputting the hip, knee, and ankle kinematics associated with each load case into the 2010 Lower Limb musculoskeletal (Arnold et al., 2010) model in OpenSim (SimTK, United States), using a published plugin (Van Arkel et al., 2013). Ligament force vectors were assumed to act in the same direction as the vector connecting their bony attachments. MRI studies of the ACL (Jordan et al., 2007) and PCL (Papannagari et al., 2007) bundles informed their force direction vectors, while the directions of the collateral ligaments with flexion were taken from a cadaveric study (Herzog and Read, 1993).

The patellofemoral joint reaction force was calculated from the predicted quadriceps load, using the relationship between patellar contact force and quadriceps force with flexion described by Ahmed et al. (1987). The location and contact area were estimated by translating and rotating the patella relative to the femur according to a kinematic dataset of the native knee (Garner et al., 2021; Dandridge et al., 2022), then identifying the contact area on the articulating surface of the femur according to descriptions given in a functional anatomical study (Goodfellow et al., 1976) and projecting the patellar contact area onto the femur. The resulting femoral contact area was then verified through comparison with MRI-measured contact areas at each flexion angle under weight-bearing conditions (Hungerford and Barry, 1979; Besier et al., 2005); deviations were less than 11%. Similar to the tibiofemoral loads, when patellofemoral loading occurred through an implant, a single point load was applied.

The resulting loads applied to the tibial and femoral models in the toe-off gait load case are described in Supplementary Tables S5, S6, respectively, as well as the stair ascent and sit-to-stand load cases in Supplementary Tables S6–S9.

### Data analysis

The percentage difference between the equivalent elastic strain in the implanted and intact bone was calculated; strain shielding (negative percentage differences) was a term used to describe regions where the strain reduced following implantation. Conversely, overstraining (positive percentage differences) was used to denote bone that experienced increased strain following implantation. Equivalent elastic strain was defined according to the Von Mises formulation, and the percentage difference in equivalent elastic strain was calculated as $\frac{\varepsilon_{equiv,implanted}-\varepsilon_{equiv,intact}}{\varepsilon_{equiv,intact}}\times 100$ .

The bone meshes were inherently different following the Boolean operations that enabled implantation. Strain data were, thus, interpolated onto the same regular grid to enable percentage difference calculation. Two interpolations were used, with grid spacing determined following a sensitivity study. First, a 2D grid with 0.1 mm spacing, using a MATLAB (MathWorks, United States) triangulation-based cubic interpolation function, was used to create visual contour maps in regions of interest. The same regions were used for all implant states, and they were defined prior to the generation of any results. For the femur: a sagittal slice through the medial condyle located at the center of the posterior fixation peg of the UKA-M, a sagittal slice through the lateral condyle at the center of the posterior fixation peg of the UKA-L, a frontal slice through the distal tail of the PFA, and a frontal slice through the condyles, at the center of the posterior fixation peg of the UKA-M. It was coincidental, yet convenient, that the surgically planned positions led to the TKA and PFA fixation pegs appearing in the condylar sagittal cross-sections. For the tibia: a frontal slice 10 mm anterior to the tibial origin that approximately bisected the ACL attachment, a transverse slice 8 mm distal to the condyles corresponding to the proximodistal midpoint of the keels of the UKA-M and UKA-L, and a transverse slice 30 mm distal to the condyles toward the distal tip of the TKA stem. Separate 2D contour plots were prepared for strain shielding (−100%–0% difference in equivalent elastic strain from intact bone) and overstraining (0%–100% difference), since this best allowed for the clear visualization of the areas of the greatest strain shielding or overstraining. In both contour plots, yellow regions ( $\geq \left|\pm 100\right|$ % difference) represented bones that experienced a strain state most different from the intact bone, while dark-blue regions represented the bone that either experienced no difference in the strain state or was overstrained when investigating strain shielding or *vice versa* ( ≤0% difference).

**FIGURE 2:**
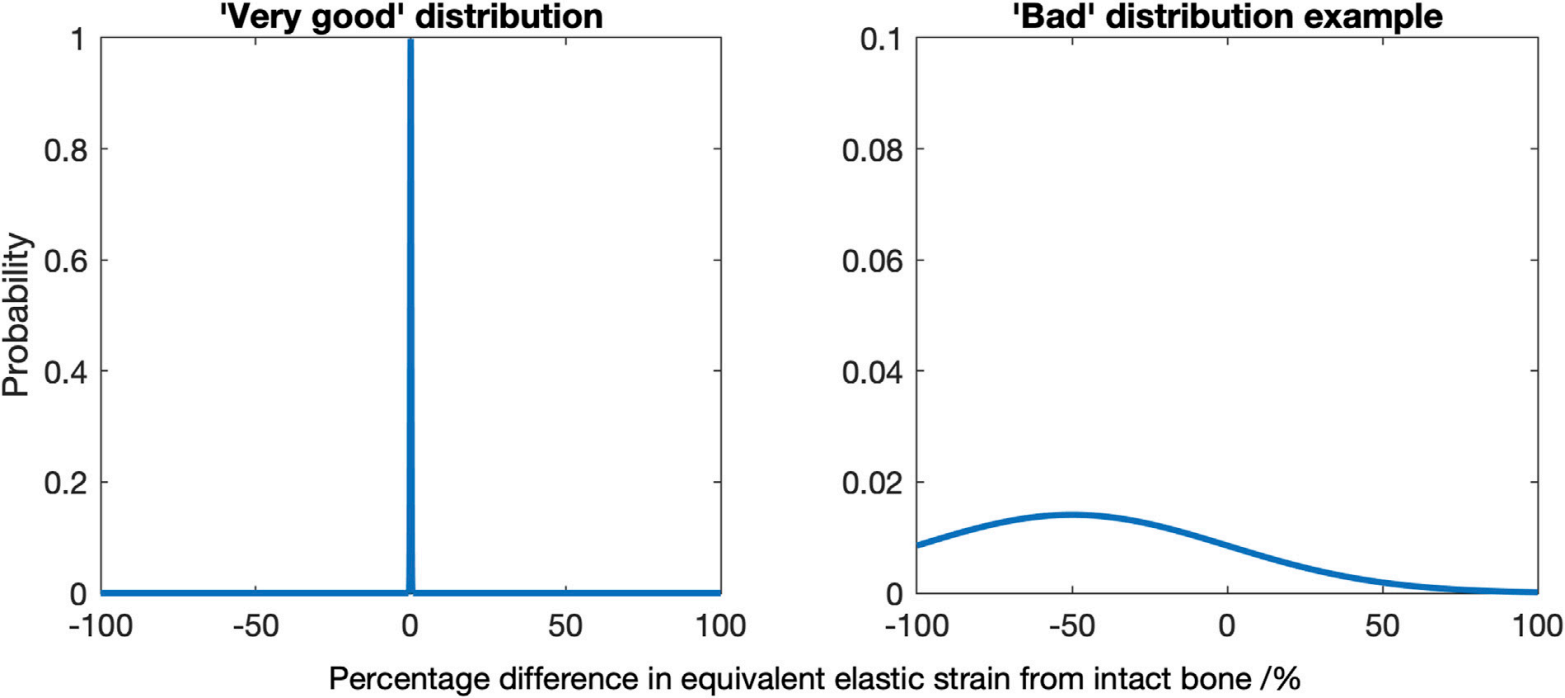
Diagrams indicating a “very good” strain difference distribution, where a mean of zero and low variance indicates most of the bone experiences near identical strains as the native bone (left), and an example of a “bad” distribution, here where there is a non-zero mean and a high variance (right).

Second, a 3D grid with 5 mm spacing, using a MATLAB triangulation-based linear interpolation function, was used to capture the strain distribution throughout the entire distal femur (1,493 sample points) and proximal tibia (1,027 sample points). This allowed a quantitative comparison for all states that was independent of the *a priori* chosen 2D slices used for visual comparison. Histograms were used quantitatively and visually to evaluate the changes in load transfer throughout the bone volume. Were there no changes in load transfer, the mean and variance would be zero. A negative mean indicates that on average, the distal femur/proximal tibia was strain-shielded, and a positive mean indicates that it was overstrained. The variance is a measure of the extent to which the load transfer was changed: a spike-like distribution with a high peak is indicative of near-native load transfer throughout the bone (low variance), whereas a wide distribution with a low peak would indicate that little of the bone experienced near-native strain and much of it was either strain-shielded or overstrained (high variance) (Figure 2). Skew indicates if there was greater spread for the strain-shielded portion of the bone or the overstrained region.

Strain energy density was also calculated and plotted as histograms. In bone remodeling algorithms, typically a “lazy zone” where no remodeling occurs is included so that only the bone experiencing changes to the mechanical stimulus over a certain threshold resulted in changes to the local bone material properties going forward. Thresholds of 50% and 75% have been used (Turner et al., 2005; Cawley et al., 2012; Dickinson, 2014). The percentage of the sample points throughout the distal femur found to exceed either threshold was, thus, calculated. A negative result implied risk of resorption, and a positive result was indicative of stimulus that encourages bone formation.

In addition to the load case analyses, sensitivity of the conclusions to a second subject-specific model (male tibia, right-sided) was also assessed. This model had also been validated previously (Tuncer et al., 2013).

## Results

### Load transfer changes

#### Strain shielding during toe-off gait

**FIGURE 3:**
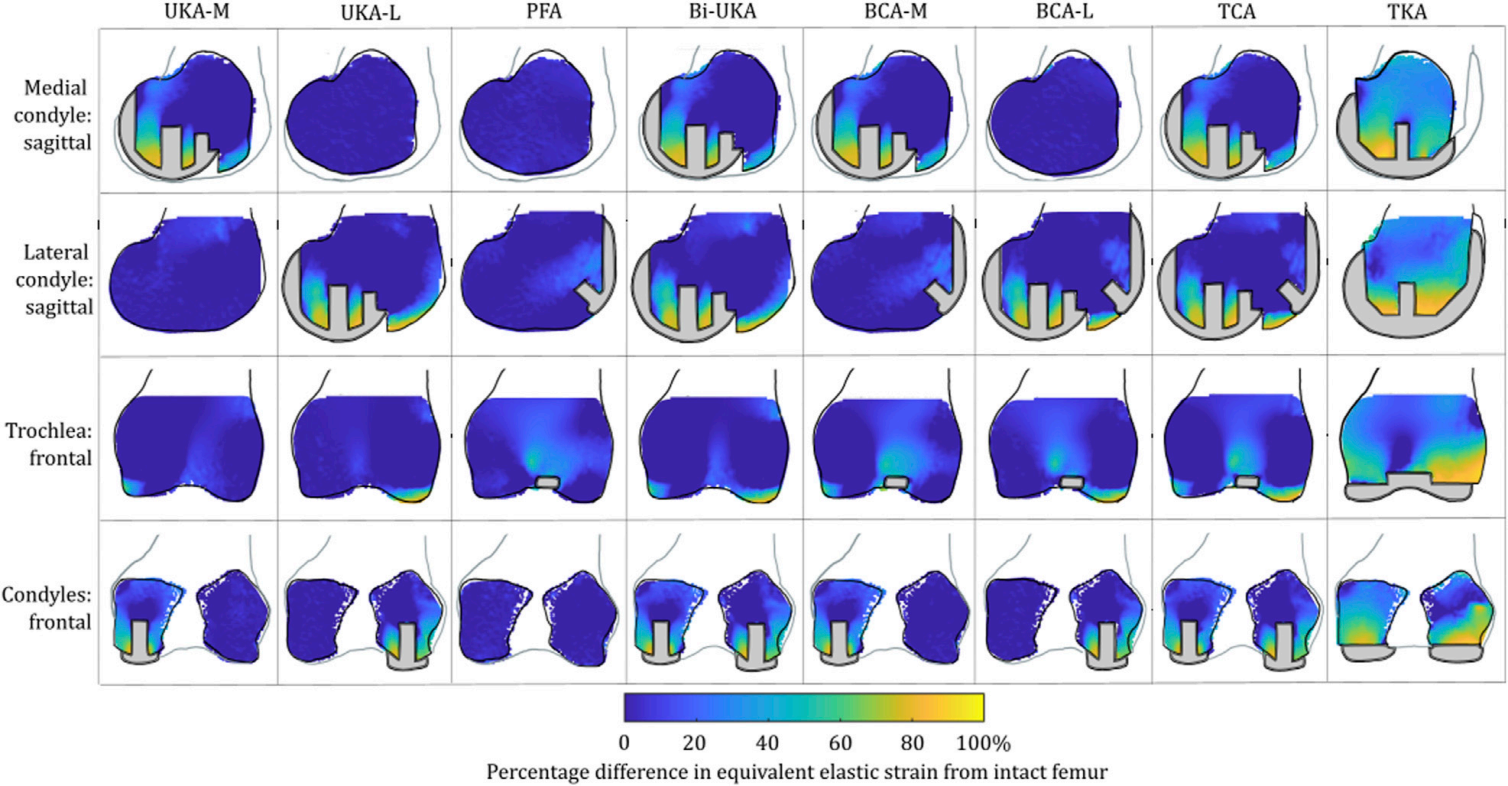
Contour plots showing the degree of strain shielding predicted during toe-off in gait, in the implanted femur compared to the intact femur. Values of strain difference have been truncated at 0% for clarity of the figure, with overstraining patterns presented separately in Figure 5.

**FIGURE 4:**
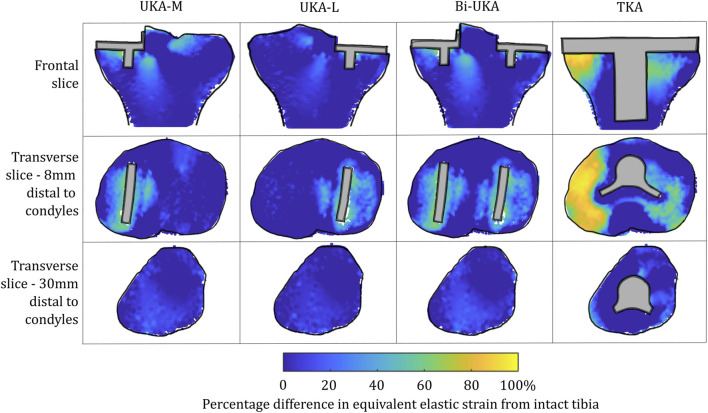
Contour plots showing the degree of strain shielding predicted during toe-off in gait, in the implanted tibia compared to the intact tibia. Values of strain difference have been truncated at 0% for clarity of the figure, with overstraining patterns presented separately in Figure 6.

For isolated PKA during toe-off in gait: UKA-M femoral strain shielding (Figure 3) was the greatest in the distal medial condyle, in the region enclosed by the implant. The same was true for the tibia (Figure 4), where strain shielding was mostly concentrated medially under the implant. Little strain shielding was observed in the femoral trochlear frontal section, the lateral condyle, or 30 mm distal to the tibial plateau, indicating near-native load transfer in these regions. The trends following UKA-L were similar but with the effects observed laterally: strain shielding was evident in the lateral condyle and plateau adjacent to the implant, with little difference in load transfer elsewhere. Strain shielding for the PFA was the greatest in the frontal slice adjacent to the implant. In the distal femur, strain shielding was greater laterally, visible in the frontal trochlear and lateral condylar slices.

Strain shielding following CPKA (Figures 3, 4) was again the greatest immediately adjacent to the implants. There were also regions where strain shielding increased between the implants (up to ∼50% strain shielding). This was not the result of an interaction leading to a new load transfer phenomenon, rather was broadly like the addition of the two isolated implant strain-shielding maps leading to regions where the strain shielding became appreciable.

With TKA, almost the entire distal femur region experienced strain shielding (Figure 3). The regions of the bone that were highly strain-shielded for PKA and CPKA were even bigger following TKA. Even when compared to TCA, where a UKA-M, UKA-L, and PFA were all implanted leaving no native articular surface, the strain shielding was appreciably increased for the monolithic TKA component. The same was true in the tibia (Figure 4); TKA strain shielding was both more intense and affected more of the tibia than for Bi-UKA. The medial and proximal regions of the tibia saw the highest degree of strain shielding, while the bone closest to the stem was not strain shielded.

#### Overstraining during toe-off gait

**FIGURE 5:**
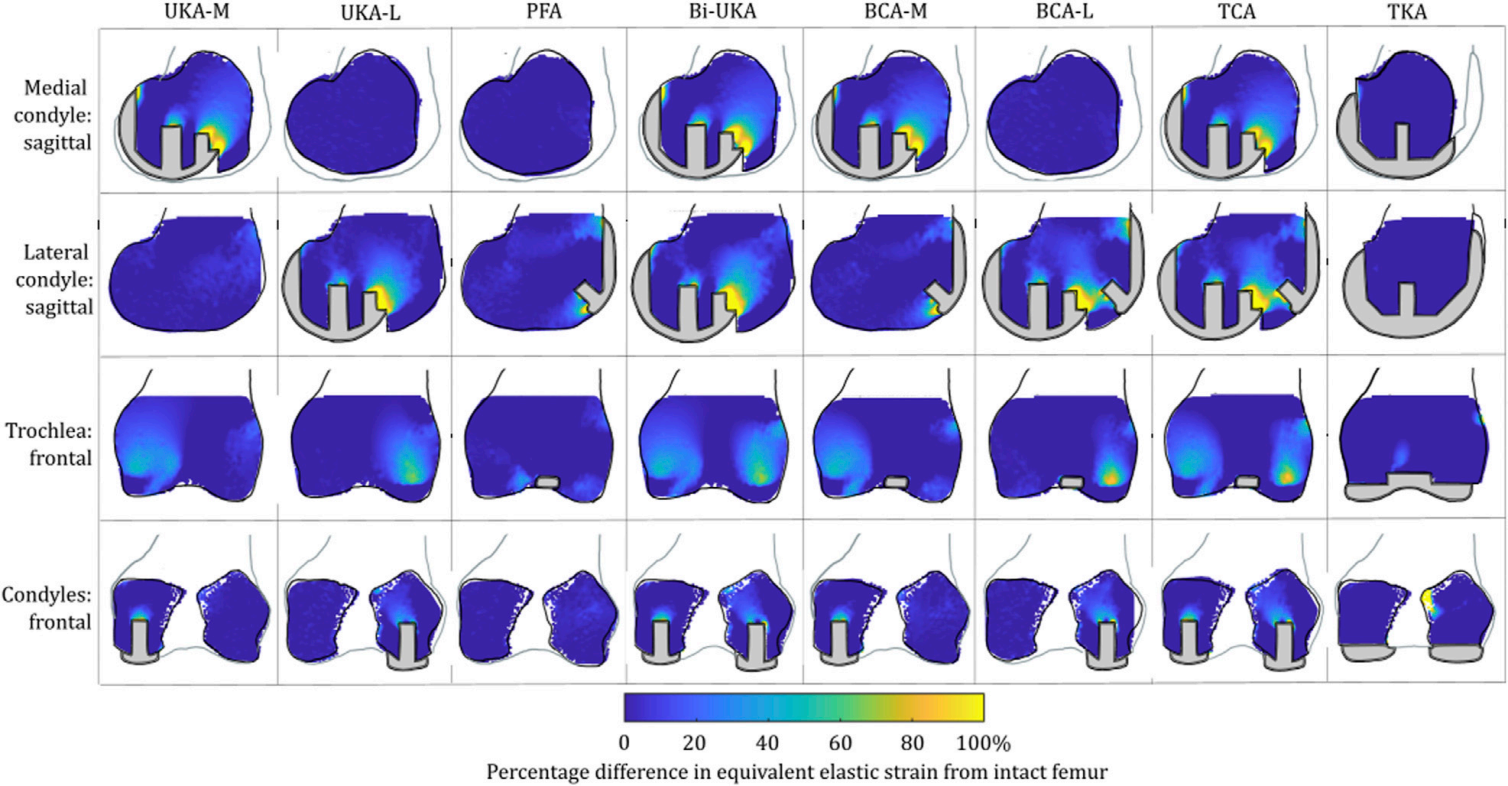
Contour plots showing the degree of overstraining predicted during toe-off in gait, in the implanted femur compared to the intact female femur. Values of strain difference have been truncated at 0% for clarity of the figure, with strain-shielding patterns presented separately in Figure 3.

**FIGURE 6:**
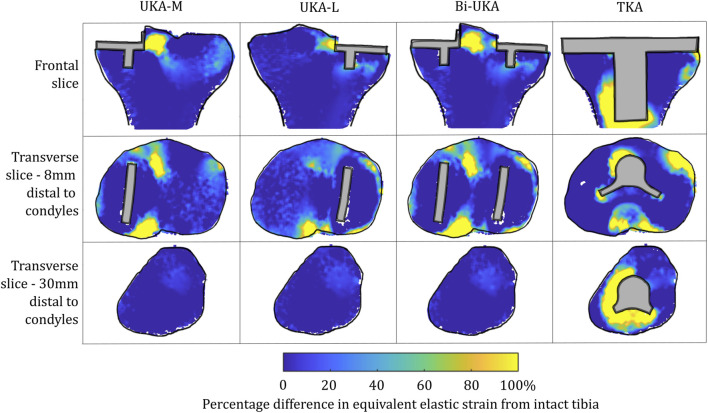
Contour plots showing the degree of overstraining predicted during toe-off in gait, in the implanted tibia compared to the intact tibia. Values of strain difference have been truncated at 0% for clarity of the figure, with strain-shielding patterns presented separately in Figure 4.

For isolated PKA during toe-off in gait: femoral overstraining (Figure 5) was the greatest for the UKA-M and UKA-L implants at the peg tips, at the most anterior tip of the implant, and at the most proximal posterior region adjacent to the implant. The result was a large region of overstraining anteriorly as these effects combined. For the tibia (Figure 6), overstraining was observed in the frontal slice lateral/medial to the UKA-M/L, respectively. Overstraining was comparatively greater for the UKA-M, suggesting interaction with the ACL under its attachment. The transverse slices 8 mm distal to the implant revealed a halo-like overstraining effect that tracked the edge of the implant. It was the greatest close to the cruciate ligaments’ attachments. For the PFA, overstraining was again evident at the tips of the implant (most proximally and most distally). A small portion of overstraining occurred around the peg, but this was less evident than that seen for the UKA-M and UKA-L.

For the CPKA procedures, overstraining increased, but as was the case for strain shielding, this was an additive effect, rather than a new load transfer phenomenon.

For TKA, no overstraining was evident in the femoral slices (Figure 5), other than on the proximomedial corner of the lateral condyle. The 3D model was inspected for evidence of overstraining not captured in these slices, and it was observed proximal and posterior to the proximal edge of the anterior flange of the TKA (similar to the overstraining observed at the anterior tip of the UKA implants that is visible in the slices shown). In the tibia (Figure 6), overstraining was predicted anterolaterally near the implant, posteriorly under the cruciate attachment, and around the stem. Overstraining around the stem was greater medially than laterally and greater distally than proximally.

#### Strain distribution during toe-off gait

**FIGURE 7:**
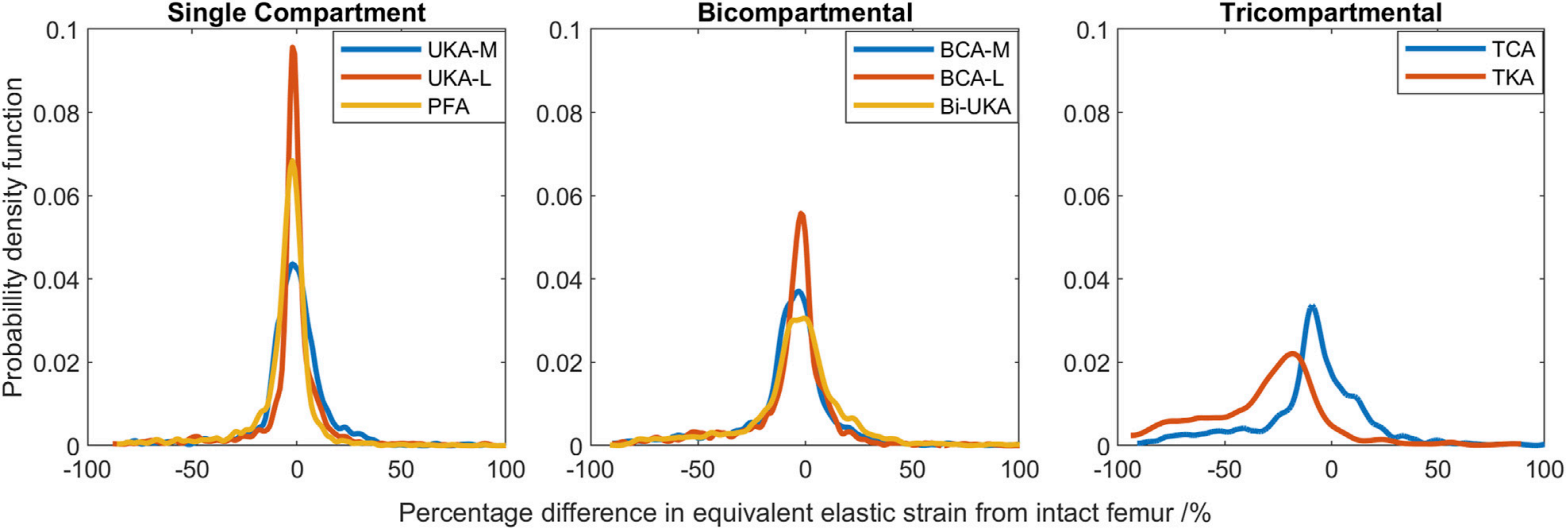
Histograms showing the distribution of strain shielding (negative x-axis) and overstraining (positive x-axis) throughout the volume of the implanted distal femur during toe-off in gait.

During toe-off in gait, the mean percentage difference in the equivalent elastic strain predicted throughout the distal femur was from −7.3% to −3.5% in the PKAs and from −8.1% to −5.2% for CPKAs affecting two compartments (Figure 7; Supplementary Table S10). In TCA, the mean strain shielding was −7.8%, while in TKA, the mean degree of strain shielding was more than three times higher, at −30%. The Bi-UKA and TKA distributions were largely symmetrical, while all the PKAs and BCA-M and BCA-L were negatively skewed, meaning that there was a wider spread of strain shielding than overstraining. The strain difference distribution was highly positively skewed for TCA. The spread of the data increased as more compartments of the knee were resurfaced; the variance $\sigma^{2}$ measured in the PKAs was roughly half that of the CPKAs affecting two compartments and almost a quarter of the spread in TKA; the trend was $\sigma_{uni}^{2}\;<\;\sigma_{bi}^{2}\;<\;\sigma_{tri}^{2}$ . The distribution in TCA was most spread; the variance was about twice the value of that in TKA.

**FIGURE 8:**
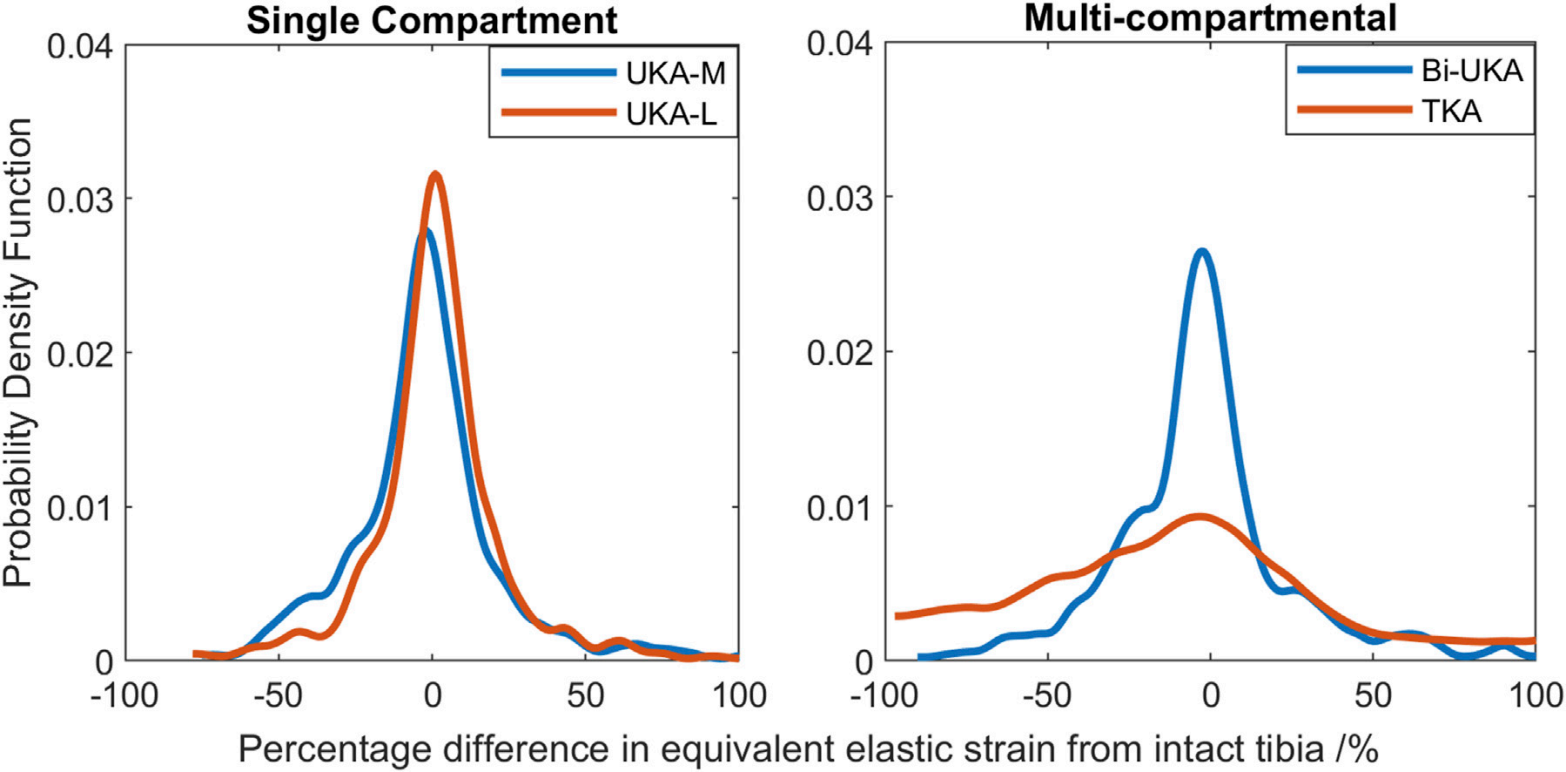
Histograms showing the distribution of strain shielding (negative x-axis) and overstraining (positive x-axis) throughout the volume of the implanted proximal tibia during toe-off in gait.

In the proximal tibia, the mean percentage difference in the equivalent elastic strain predicted was positive for all arthroplasties considered, indicating overstraining rather than strain shielding (Figure 8; Supplementary Table S10). All arthroplasties showed positive skew. The mean percentage difference in the equivalent elastic strain predicted was from 0.4% to 6.4% for the CPKAs, while it was much higher, at 21.3%, for TKA. The variance in TKA was also about ten times greater than in CPKA.

#### Sensitivity to the load case

Results for the PKA and CPKA implant states were largely insensitive to the load case. Similar patterns of strain shielding and overstraining were predicted during stair ascent and sit-to-stand: strain shielding occurred between pegs and in the offloaded anterior notch in the femur, and overstraining was predicted at the tips of pegs (Supplementary Figures S1–S4, S7–S10). Differences were seen according to the mediolateral condylar loading ratio and the direction of the condylar load application on the femur, which affected the direction in which the regions of strain shielding and overstraining dissipated around features.

**FIGURE 9:**
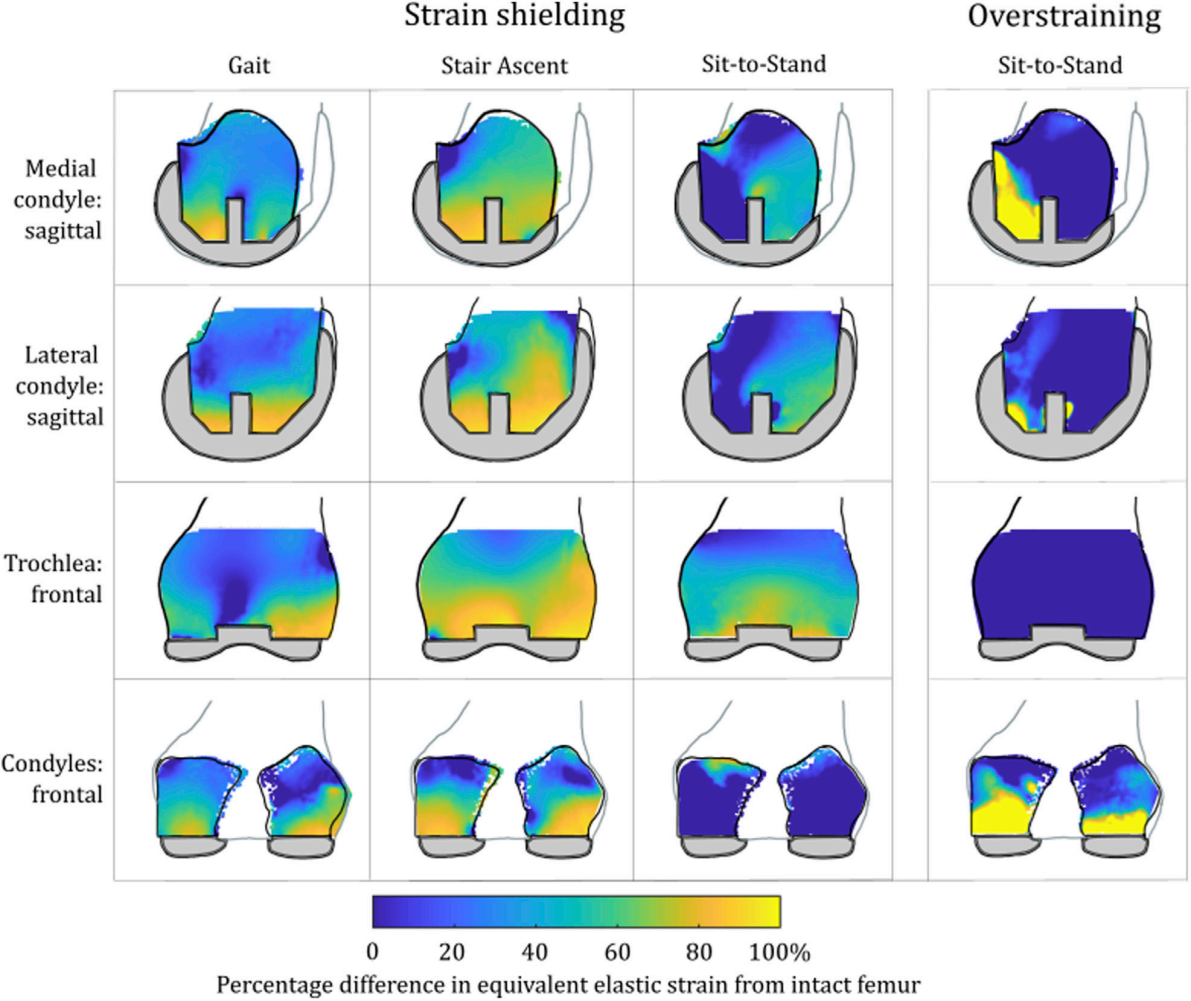
Contour plots showing the degree of strain shielding predicted during toe-off gait (left), stair ascent (middle, left), and sit-to-stand (middle, right) for the femur implanted with TKA, as well as overstraining associated with sit-to-stand (right).

There were notable differences in the load transfer associated with each load case in the femoral TKA model (Figure 9). For both the lower-flexion gait and stair ascent load cases, almost the whole volume of the distal femur investigated experienced strain shielding, while in contrast to this, at the higher flexion angle during sit-to-stand, the posterior femoral condyles, instead, experienced a high degree of overstraining.

#### Strain energy density changes

**FIGURE 10:**
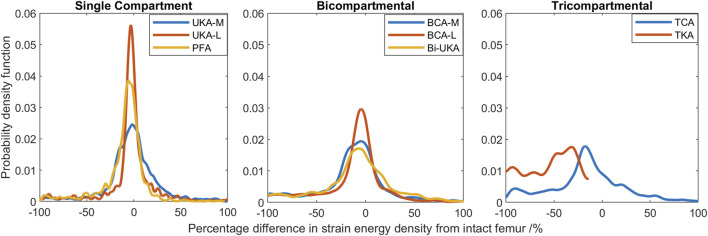
Histograms showing the distribution of changes in strain energy density predicted throughout the volume of the implanted distal femur during toe-off in gait.

Compared to the results for the same load case with the change in equivalent elastic strain considered (Figure 7), the general trends observed for strain energy density are the same (Figure 10); PKA resulted in the tallest and narrowest distributions, while TCA and TKA had the flattest and widest change in strain energy density distributions. In addition, similar to the equivalent elastic strain results, strain energy density distributions were largely symmetrical for all PKA and CPKA variants. For TKA, however, almost all the changes in strain energy density were negative.

**FIGURE 11:**
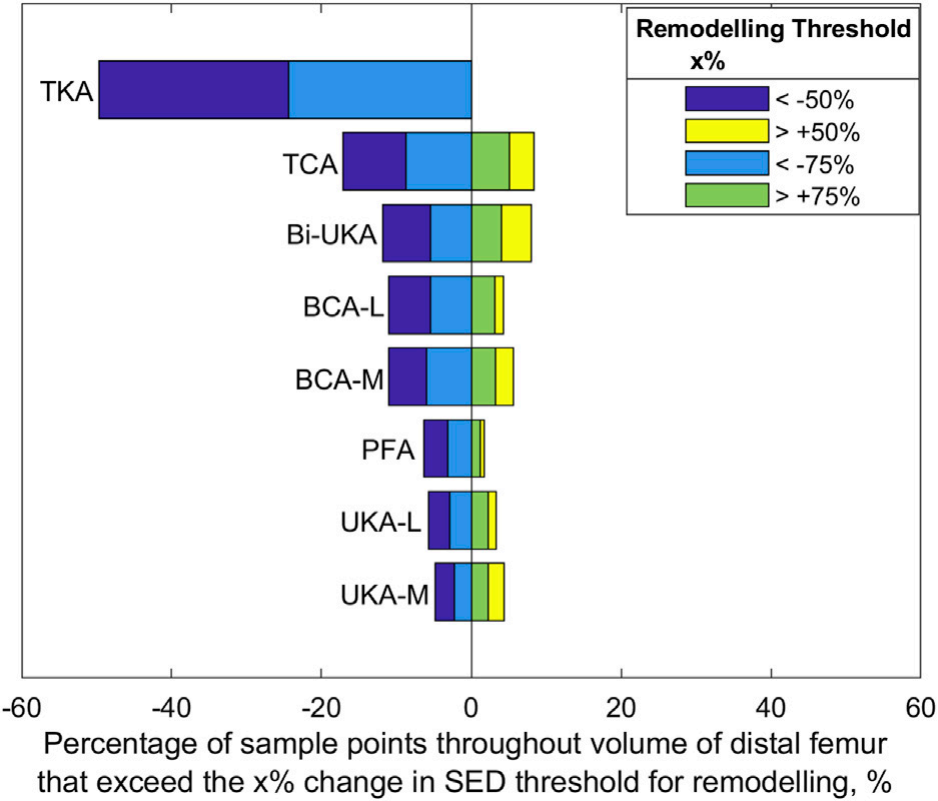
Bar chart showing the percentage of sample points in the distal femur found to exceed a change SED stimulus threshold of 50% and 75% at toe-off in gait.

Given a remodeling stimulus threshold of 75%, roughly 3% of the sample points in the distal femur implanted with only PKA were predicted to experience a mechanical stimulus causing bone resorption, while this was between 5% and 9% for CPKA and 24% for TKA. With a lower threshold of 50%, the percentage of sample points that would be at risk of bone resorption in the immediate post-operative period rose to 50% in TKA (Figure 11).

#### Sensitivity to a second subject-specific tibial FE model

**FIGURE 12:**
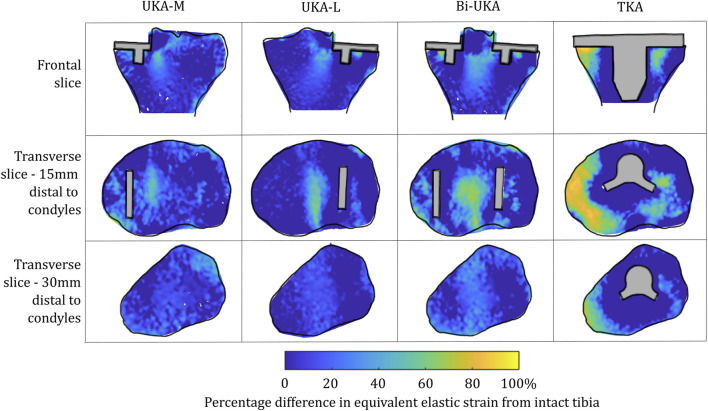
Contour plots showing the degree of strain shielding predicted during toe-off in gait, in the implanted male tibia compared to the intact male tibia.

Similar strain-shielding patterns were observed in the male tibia (Figure 12) as in the female tibia. The biggest difference observed between the tibial results were in the mid-keel transverse slice of the Bi-UKA case and UKA-L, where a region of about 60% strain shielding was predicted in between the two implants, under the tibial eminence in the male model.

**FIGURE 13:**
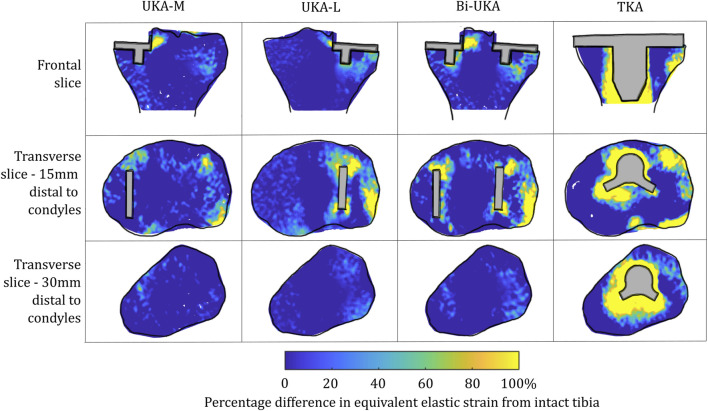
Contour plots showing the degree of overstraining predicted during toe-off in gait, in the implanted male tibia compared to the intact male tibia.

Similar patterns and magnitudes of overstraining were also observed for all the arthroplasties modeled (Figure 13: frontal and distal transverse slices). The most notable differences were predicted in the most proximal transverse slice investigated, in UKA-M, UKA-L, and Bi-UKA, when compared to the results of the female model.

**FIGURE 14:**
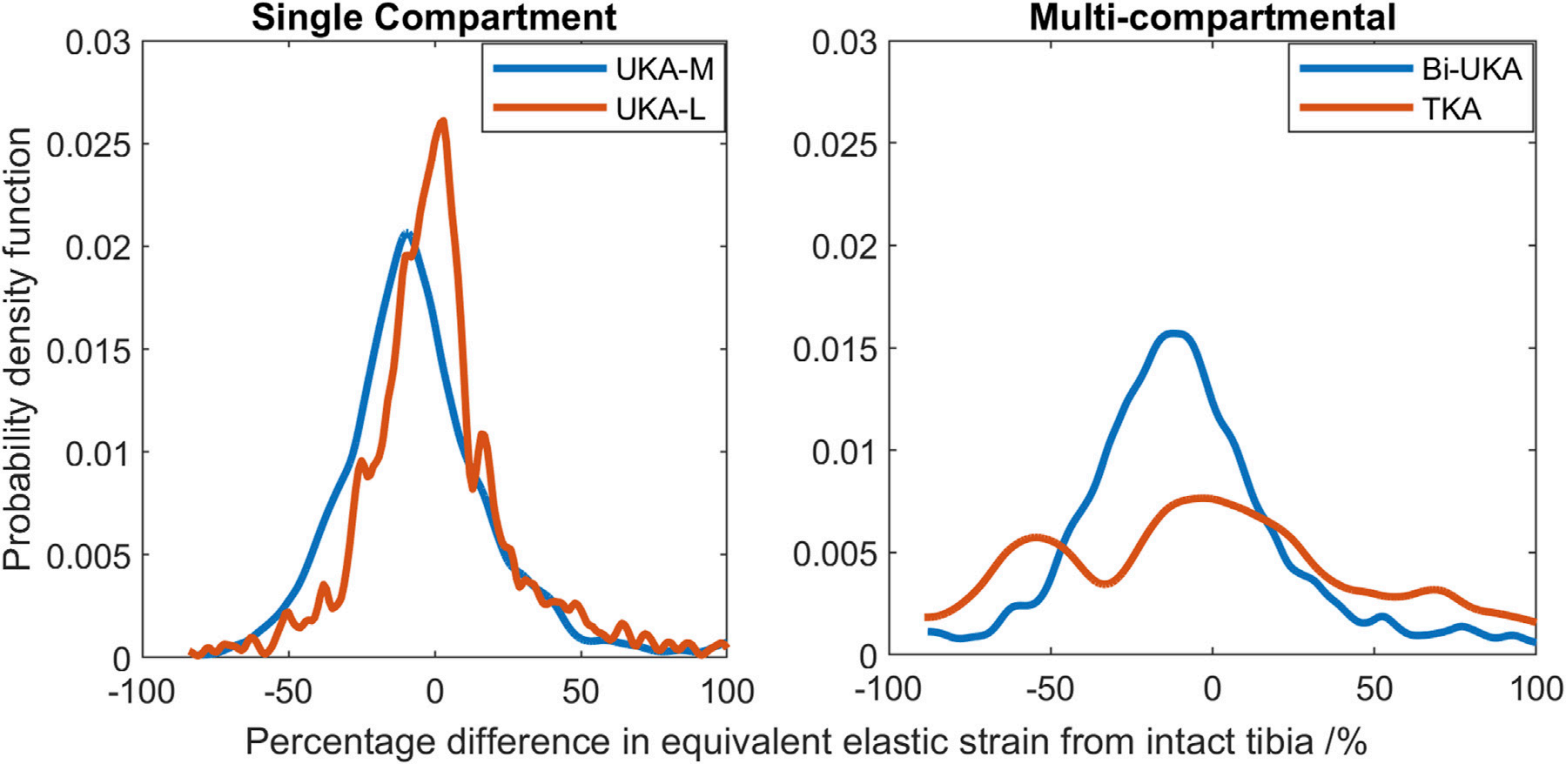
Histograms showing the distribution of strain shielding (negative x-axis) and overstraining (positive x-axis) throughout the volume of the implanted proximal male tibia during toe-off in gait.

The PKAs in the male tibia had the least-disturbed distributions, with the mean difference close to zero, and low variance (Figure 14). As more bone was removed in Bi-UKA and TKA, respectively, the distribution flattened as the variance increased, and more of the proximal tibia experienced progressively large changes to the native strain. Thus, the trends observed for the female bone were replicated.

## Discussion

This study showed, for the first time, that TKA leads to mean strain shielding more than three times higher than for partial and multi-compartmental knee arthroplasty. For all eight implant combinations analyzed, the load was found to have transferred from within the implant’s bone footprint to the rim of the implant and the tips of any peg/keel-like features. In TKA, the pegs, keel, and implant footprint are larger, and hence, more of the bone is shielded. The monolithic components required for TKA are, thus, doubly disadvantageous for bone biomechanics, requiring greater initial bone resection and having a larger negative effect on bone remodeling stimulus, disrupting load transfer more than any compartmental arthroplasty alternative (Figures 3–8). This change in remodeling stimulus is important as for every one standard deviation decrease in BMD, the relative risk of fracture increases by 2.6% (Prince et al., 2019).

**FIGURE 15:**
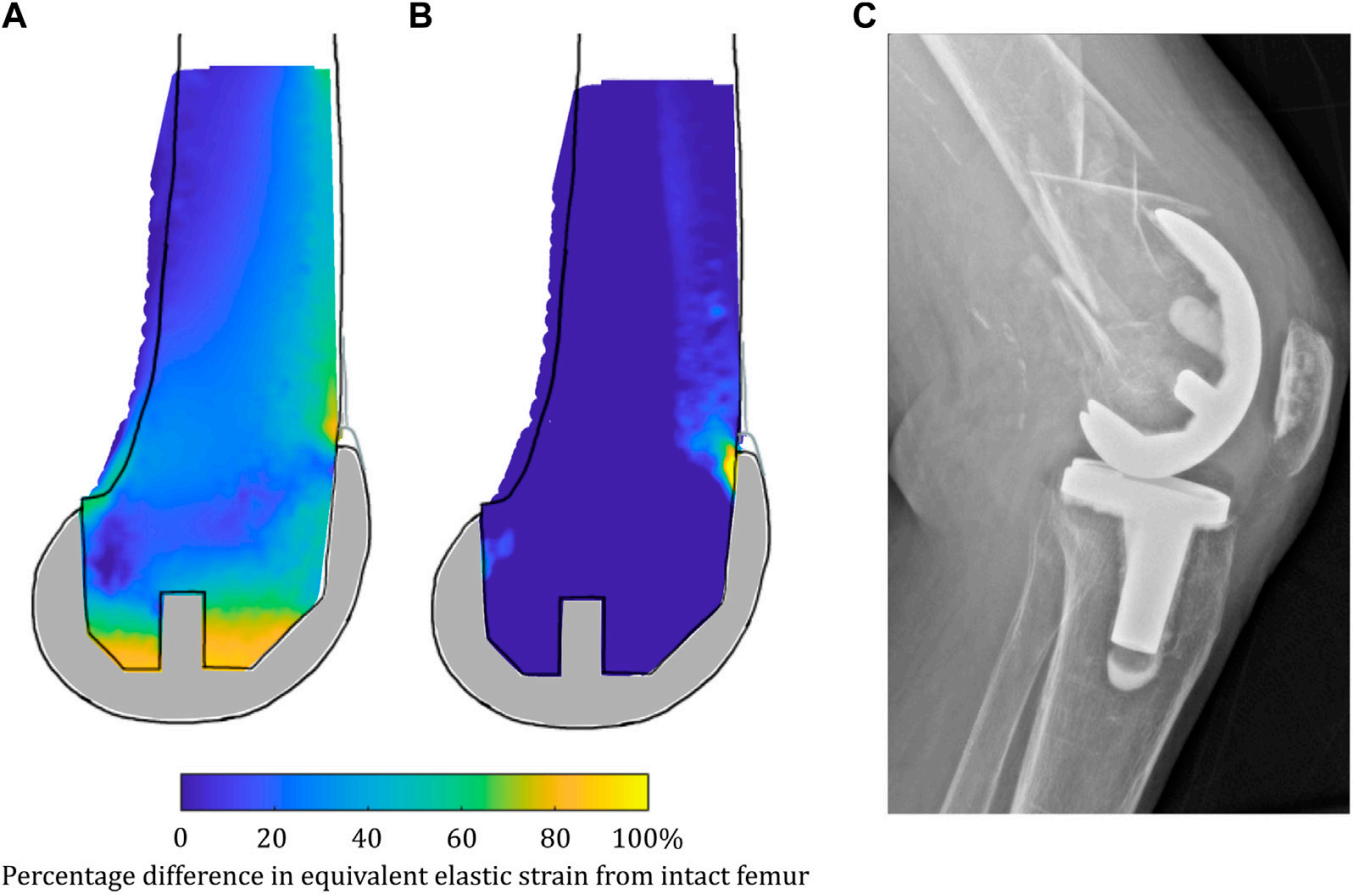
**(A)** Strain shielding during low-flexion gait combined with **(B)** overstraining during higher-flexion stair ascent in a sagittal slice through the lateral femoral condyle when implanted with TKA may contribute to periprosthetic femoral fractures such as that shown in **(C)** a lateral radiograph of a femoral TKA periprosthetic fracture.

Clinically, a meta-analysis of changes in distal femoral BMD after TKA (Prince et al., 2019) found that there was a non-recoverable and rapid decrease in BMD following TKA of 17.5% in the intracondylar regions that was maintained at 2 years. The mean strain shielding in this region in our model was predicted to be between −10.5% and −42.6% (Supplementary Table S10), and the contour plots indicated gross strain shielding throughout the distal femur (Figure 9), thus agreeing with clinical trends. The sit-to-stand load case, for which the condylar region was found to be overstrained rather than strain-shielded, suggests high-flexion activity may help protect bone stock following TKA. This may help explain why complete bone loss is not observed clinically despite the gross strain shielding predicted for stair climbing/gait. Other FE and DEXA studies have also indicated BMD loss in the femur behind the anterior flange in TKA (Petersen et al., 1995; van Lenthe et al., 1997) and PFA (Van Jonbergen et al., 2010), which also agree with our findings. In our analyses, the load was found to have transferred from the condylar region to proximal to the implant, where there were high levels of overstraining predicted. When considering a proximally extended sagittal slice through the lateral femoral condyle, a further observation was made from our results: in low-flexion activities (gait), the entire femoral slice was strain-shielded when implanted with TKA, with higher-intensity strain shielding predicted just proximal to the anterior flange of the implant (Figure 15A), which, in the long term, would be expected to weaken the bone proximal to the implant. However, when higher-flexion activities (stair ascent) were modeled, this same region of bone proximal to the implant was predicted to be highly overstrained (Figure 15B). Rorabeck type II fractures (Figure 15C) have been found to be the most common type of periprosthetic fracture associated with TKA (Ebraheim et al., 2015), and their typical location coincides with the predicted overstrained region. Joint registries do not currently record many such periprosthetic fractures as causing revision, as they are typically treated with long locking plates and the TKA implants remaining *in situ*; however, such fractures are associated with substantial morbidities such as non-union and infection (Ebraheim et al., 2015).

Femoral BMD loss has also been measured in the first 3 months after UKA-M (Soininvaara et al., 2013; Tuncer et al., 2013). Our strain-shielding predictions (Figure 3) correlated with the clinical finding that the posterior region of interest had the largest reduction in BMD.

Tibial BMD following TKA has been suggested to be dependent on the nature of the preoperative varus–valgus deformity that was corrected. For a preoperative varus deformity where the medial compartment was overloaded, its correction would act to unload it and a relative decrease in BMD has been observed after arthroplasty (Deen et al., 2018; Yoon et al., 2018). The converse effect has been observed in the lateral compartment. In our study, the load case sensitivity analyses found that the mediolateral condylar loading ratio and the direction of the condylar load application on the femur affected the predicted strain shielding, agreeing with these clinical observations. Dudhniwala et al. (2016) investigated radiolucencies in 15 patients, 6 months and 1 year following monobloc BCA-M. Femoral comparisons are not appropriate as we did not model a monobloc femoral component. In the tibia, however, they found that the medial portion of the resurfaced medial tibial condyle had a reduced bone density both anteriorly and posteriorly, immediately under the tibial tray, agreeing with our prediction of strain shielding in this region (Figure 4). Others have found an increase in BMD in the medial tibial metaphysis 3 months and 7 years post-operatively (Soininvaara et al., 2013). This correlated with the positive mean of the strain difference distributions predicted for each load case in the proximal tibia (Supplementary Table S10). While classically, TKA has been associated with supracondylar femoral fractures (Parvizi et al., 2008), UKA has been associated with tibial condylar fracture (Thoreau et al., 2022). Although not explicitly investigated in this study, the areas of high overstraining predicted in the tibia, namely, adjacent to the sagittal wall or under the keel of a unicondylar prosthesis, correlate with the observed fracture initiation points (Burger et al., 2022).

To our knowledge, this is the first study to present mechanical results such as bone strain differences in a volume as a histogram. This analysis approach enabled quantitative comparison across the entire bone volume, with metrics that are less sensitive than maximum stress/strain/strain energy density. It also reduces measurement bias associated with only considering regions of interest. Others have analyzed strain shielding in the lab (Correa et al., 2018) or with FE analysis (Pal et al., 2010; Chanda et al., 2015). In the lab, Completo et al. measured cortical strains at toe-off (Completo et al., 2008) and found a 65% difference following TKA, agreeing with the 60%–80% strain shielding predicted on the medial posterior cortex under the tibial tray in our FE models (Figure 4: proximal transverse slice). Their posterior cortex distal findings (20% strain shielding) also agree with our findings. However, they found 10% strain shielding anteromedially, whereas in our model, overstraining was predicted. This difference is likely due to the use of sawbones versus cadaveric tibiae and the boundary conditions where the *in vitro* experiment did not consider muscular or ligamentous contributions. An experimental analysis of strain differences following patellofemoral arthroplasty (Meireles et al., 2010) used strain gauges at locations roughly corresponding to points on the perimeter of the frontal slice (Figure 5). They found −43.5% to −67.5% strain shielding and −15.7% to −28.7% in the lateral and medial condyles, respectively. These data agree broadly with our predictions of 40% and 20% laterally/medially, respectively, during sit-to-stand (Supplementary Figure S7). During gait, however, near the medial epicondyle, they measured around 20% overstraining, while our model predicted close to no difference; this may be due to the absence of the medial collateral ligament from their model, which attaches at the medial epicondyle. Our models predicted increased strain shielding in the lateral distal femur in comparison to in the medial condyle. This was likely a combination of the more lateral position of the trochlear component and the increased loading on the lateral trochlea in the native knee because of the quadriceps angle. The phenomenon of remodeling-driven bone loss has been studied both in terms of strain and stress shielding. We chose to study the differences in strains developed pre- and post-implantation since bone remodeling in terms of triggering osteocyte activity has been measured to be controlled by changes in vascular and lacunar pore pressures associated with macroscopic bone strains (Scheiner et al., 2016). Further to this, the strain-related measure of strain energy density is the most commonly used mechanical stimulus when FE-based iterative bone remodeling schemes are employed. Contextualizing our results against FE studies that looked at stress shielding post-TKA, Cawley et al. predicted increased stress at the distal stem of the tibial component and stress shielding just distal to the tray (Cawley et al., 2012). This agrees with the locations within the tibia that we predicted to be overstrained or strain-shielded with TKA. An FE study investigating the stress shielding associated with PFA found some stress shielding posterior to the trochlear implant in PFA, similar to the strain shielding we predicted, but similarly predicted much higher stress shielding in the same area for TKA (van Jonbergen et al., 2012).

This study’s strength lies in the number of models studied (nine femoral and five tibial), for female tibia, each with three load cases, and an additional five male tibial models. This is the first study to analyze the effects of all eight types of knee arthroplasty in the same modeling framework and with the bone models with shared open access to enable others to build on our research. There was a limitation in the development of these load cases, however, as they required mixing data from multiple sources for which there are inevitably assumptions. For example, it was assumed that TKA tibiofemoral joint reaction force data are relevant to PKA and CPKA, or that ligament forces estimated for the native knee are transferable to a PKA implanted knee. This may not be the case as differences in gait kinetics and kinematics have been observed; for example, UKA patients have been observed to walk faster than TKA patients (Wiik et al., 2013; Jones et al., 2016; Agarwal et al., 2019), as have patients with Bi-UKA (Garner et al., 2021c) and BCA-M (Garner et al., 2021e); however, the muscle loads were not changed to account for this. While these assumptions did not prevent our data replicating known clinical trends, or from correlating with prior laboratory work, there are likely subtle impacts that may have occurred. For example, in Rasnick et al.’s musculoskeletal models of healthy and TKA subjects climbing stairs, they predicted much lower quadriceps forces during weight acceptance for the TKA subjects, but higher forces produced by the knee flexors, in an apparently compensatory strategy (Rasnick et al., 2016). This may mean that the FE models have under-predicted the degree of strain shielding posterior to the anterior flange of the femoral TKA component and, correspondingly, might have under-predicted the degree of overstraining in the posterior condyles close to the gastrocnemii attachments during stair ascent. This study was also limited as we only looked at relative measures of strain, comparing the pre- and post-implantation strain states, and did not investigate absolute strain values. As such, no direct assessment of fracture risk by the comparison of peak strains to bone yield strains can be made, though the regions of high overstraining identified may correlate with periprosthetic fractures observed clinically. Detailed work using the finite element method to investigate the fracture risk associated with UKA-M has been conducted previously (Sawatari et al., 2005; Dai et al., 2018; Pegg et al., 2020). Additionally, previous work by the present authors has investigated the risk of intraoperative tibial eminence avulsion fracture associated with Bi-UKA, finding that relative implant positioning was an important risk factor (Stoddart et al., 2022). There is still room for further work looking at the femoral fracture risk associated with CPKA. Another limitation was that when comparing the results to that of the additional second tibial model, the natural variations in anatomy meant it was difficult to find equivalent slices to compare the models. The male tibia was larger than the female tibia, so using the same distance would not necessarily imply that the equivalent part of bone was being considered. Efforts were made to use equivalent slice locations, for example, by choosing a different depth distal to the tibial plateau for the most proximal transverse slice, approximately halfway down the length of the implant keels. The histogram data were, thus, useful for these comparisons, with the male/female trends matching well. Finally, remodeling algorithms can be used to predict how the mechanical stimulus (strain shielding or change in strain energy density) leads to changes in BMD, as has been demonstrated by others (Pérez et al., 2010; Phillips et al., 2015; Mathai et al., 2022). In this study, we prioritized direct comparison of all the arthroplasty types, for different load cases and different anatomies, over a more detailed remodeling analysis of one or two variants. Our data are, thus, best interpreted as the stimulus for likely remodeling in the early post-operative period, and the resulting data reflect known clinical trends for the early post-operative period well (Petersen et al., 1995; Van Jonbergen et al., 2010; Prince et al., 2019). Only one brand (Zimmer Biomet) of implants was considered, so variations in geometries and materials used in other products may affect the results.

## Conclusion

Arthroplasty inherently disturbs load transfer to bone around the knee joint; however, less is better when it comes to bone: PKA and CPKA lead to more normal load transfer than TKA, even when all articulating surfaces are replaced during tricompartmental arthroplasty.

The intact bone models used for this research are provided as open access Supplementary Materials to facilitate future research at other centers. The histogram analyses presented here are recommended as a quantitative way to compare multiple tests without risk of inadvertently excluding key data. The sensitivity of the results to load case highlights the need to consider multiple load cases in any future analyses using these models.

## Data Availability

The datasets presented in this study can be found in online repositories. The names of the repository/repositories and accession number(s) can be found in the article/Supplementary Material.

## References

[B1] Agarwal A. Miller S. Hadden W. Johnston L. Wang W. Arnold G. (2019). Comparison of gait kinematics in total and unicondylar knee replacement surgery. Ann. R. Coll. Surg. Engl. 101, 391–398. 10.1308/rcsann.2019.0016 31155888 PMC6554568

[B2] Ahmed A. M. Burke D. L. Hyder A. (1987). Force analysis of the patellar mechanism. J. Orthop. Res. 5, 69–85. 10.1002/jor.1100050110 3819912

[B3] Anijs T. Eemers S. Minoda Y. Wolfson D. Verdonschot N. Janssen D. (2022). Computational tibial bone remodeling over a population after total knee arthroplasty: a comparative study. J. Biomed. Mat. Res. - Part B Appl. Biomater. 110, 776–786. 10.1002/jbm.b.34957 PMC9297982 34661334

[B4] Arnold E. M. Ward S. R. Lieber R. L. Delp S. L. (2010). A model of the lower limb for analysis of human movement. Ann. Biomed. Eng. 38, 269–279. 10.1007/s10439-009-9852-5 19957039 PMC2903973

[B5] Bergmann G. Bender A. Graichen F. Dymke J. Rohlmann A. Trepczynski A. (2014). Standardized loads acting in knee implants. PLoS One 9, e86035. 10.1371/journal.pone.0086035 24465856 PMC3900456

[B6] Besier T. F. Draper C. E. Gold G. E. Beaupré G. S. Delp S. L. (2005). Patellofemoral joint contact area increases with knee flexion and weight-bearing. J. Orthop. Res. 23, 345–350. 10.1016/j.orthres.2004.08.003 15734247

[B7] Biazzo A. Manzotti A. Confalonieri N. (2018). Bi-unicompartmental versus total knee arthroplasty: long term results. Acta Orthop. belg. 84, 237–244. 30840563

[B8] Brittain R. Howard P. Lawrence S. Stonadge J. Wilkinson M. Wilton T. (2021). The national joint registry 18th annual report 2021. London: National Joint Registry. 35072993

[B9] Burger J. A. Jager T. Dooley M. S. Zuiderbaan H. A. Kerkhoffs G. M. M. J. Pearle A. D. (2022). Comparable incidence of periprosthetic tibial fractures in cementless and cemented unicompartmental knee arthroplasty: a systematic review and meta-analysis. Knee Surg. Sport. Traumatol. Arthrosc. 30, 852–874. 10.1007/s00167-021-06449-3 PMC8901491 33528591

[B10] Cawley D. T. Kelly N. Simpkin A. Shannon F. J. McGarry J. P. (2012). Full and surface tibial cementation in total knee arthroplasty: a biomechanical investigation of stress distribution and remodeling in the tibia. Clin. Biomech. 27, 390–397. 10.1016/j.clinbiomech.2011.10.011 22079691

[B11] Chanda S. Dickinson A. Gupta S. Browne M. (2015). Full-field *in vitro* measurements and *in silico* predictions of strain shielding in the implanted femur after total hip arthroplasty. Proc. Inst. Mech. Eng. Part H. J. Eng. Med. 229, 549–559. 10.1177/0954411915591617 26112349

[B12] Completo A. Fonseca F. Simões J. A. (2008). Strain shielding in proximal tibia of stemmed knee prosthesis: experimental study. J. Biomech. 41, 560–566. 10.1016/j.jbiomech.2007.10.006 18036530

[B13] Correa T. A. Pal B. van Arkel R. J. Vanacore F. Amis A. A. (2018). Reduced tibial strain-shielding with extraosseous total knee arthroplasty revision system. Med. Eng. Phys. 0, 22–28. 10.1016/j.medengphy.2018.09.006 PMC6236098 30314902

[B14] Dai X. Fang J. Jiang L. Xiong Y. Zhang M. Zhu S. (2018). How does the inclination of the tibial component matter? A three-dimensional finite element analysis of medial mobile-bearing unicompartmental arthroplasty. Knee 25, 434–444. 10.1016/j.knee.2018.02.004 29685499

[B15] Dandridge O. Garner A. Amis A. A. Cobb J. P. van Arkel R. J. (2022). Variation in the patellar tendon moment arm identified with an improved measurement framework. J. Orthop. Res. 40, 799–807. 10.1002/jor.25124 34191354

[B16] Deen J. T. Clay T. B. Iams D. A. Horodyski M. Parvataneni H. K. (2018). Proximal tibial resorption in a modern total knee prosthesis. Arthroplast. Today 4, 244–248. 10.1016/J.ARTD.2017.10.005 29896562 PMC5994597

[B17] Dickinson A. S. (2014). Activity and loading influence the predicted bone remodeling around cemented hip replacements. J. Biomech. Eng. 136, 041008. 10.1115/1.4026256 24337038

[B18] Dudhniwala A. G. Rath N. K. Joshy S. Forster M. C. White S. P. (2016). Early failure with the Journey-Deuce bicompartmental knee arthroplasty. Eur. J. Orthop. Surg. Traumatol. 26, 517–521. 10.1007/s00590-016-1760-4 27001223

[B68] Ebraheim N. A. Kelley L. H. Liu X. Thomas I. S. Steiner R. B. Liu J. (2015). Periprosthetic distal femur fracture after total knee arthroplasty: a systematic review. Orthop. Surg. 7 (4), 297–305. 10.1111/os.12199 26790831 PMC6583744

[B19] Garner A. J. Dandridge O. W. Amis A. A. Cobb J. P. van Arkel R. J. (2021b). Bi-unicondylar arthroplasty: a biomechanics and clinical outcomes study. Bone Jt. Res. 10, 723–733. 10.1302/2046-3758.1011.bjr-2021-0151.r1 PMC8636181 34761697

[B20] Garner A. J. Dandridge O. W. Amis A. A. Cobb J. P. van Arkel R. J. (2021c). Bi-unicondylar arthroplasty. Bone Jt. Res. 10, 723–733. 10.1302/2046-3758.1011.BJR-2021-0151.R1 PMC8636181 34761697

[B21] Garner A. J. Dandridge O. W. Amis A. A. Cobb J. P. van Arkel R. J. (2021d). Partial and combined partial knee arthroplasty: greater anterior-posterior stability than posterior cruciate–retaining total knee arthroplasty. J. Arthroplasty 36, 3765–3772.e4. 10.1016/j.arth.2021.06.025 34330602

[B22] Garner A. J. Dandridge O. W. van Arkel R. J. Cobb J. P. (2021e). Medial bicompartmental arthroplasty patients display more normal gait and improved satisfaction, compared to matched total knee arthroplasty patients. Knee Surg. Sport. Traumatol. Arthrosc. 31, 830–838. 10.1007/s00167-021-06773-8 PMC9958162 34689224

[B23] Garner A. J. Dandridge O. W. van Arkel R. J. Cobb J. P. (2021f). The compartmental approach to revision of partial knee arthroplasty results in nearer-normal gait and improved patient reported outcomes compared to total knee arthroplasty. Knee Surg. Sport. Traumatol. Arthrosc. 31, 1143–1152. 10.1007/s00167-021-06691-9 PMC9957906 34415369

[B24] Garner A. Dandridge O. Amis A. A. Cobb J. P. van Arkel R. J. (2021a). The extensor efficiency of unicompartmental, bicompartmental, and total knee arthroplasty. Bone Jt. Res. 10, 1–9. 10.1302/2046-3758.101.BJR-2020-0248.R1 PMC7845459 33380175

[B25] Garner A. Van Arkel R. J. Cobb J. (2019). Classification of combined partial knee arthroplasty. Bone Jt. J. 101 B, 922–928. 10.1302/0301-620X.101B8.BJJ-2019-0125.R1 PMC6681677 31362558

[B26] Goodfellow J. Hungerford D. S. Zindel M. (1976). Patello-femoral joint mechanics and pathology. 1: functional anatomy of the patello-femoral joint. J. Bone Jt. Surg. 58-B, 287–290. 10.1302/0301-620x.58b3.956243 956243

[B27] Herzog W. Read L. J. (1993). Lines of action and moment arms of the major force-carrying structures crossing the human knee joint. J. Anat. 182, 213–230. 8376196 PMC1259832

[B28] Huiskes R. Weinans H. Grootenboer H. J. Dalstra M. Fudala B. Slooff T. J. (1987). Adaptive bone-remodeling theory applied to prosthetic-design analysis. J. Biomech. 20, 1135–1150. 10.1016/0021-9290(87)90030-3 3429459

[B29] Hume D. R. Navacchia A. Rullkoetter P. J. Shelburne K. B. (2019). A lower extremity model for muscle-driven simulation of activity using explicit finite element modeling. J. Biomech. 84, 153–160. 10.1016/j.jbiomech.2018.12.040 30630624 PMC6361714

[B30] Hungerford D. S. Barry M. (1979). Biomechanics of the patellofemoral joint. Clin. Orthop. Relat. Res. 144, 9–15. 10.1097/00003086-197910000-00003 535256

[B31] Jones G. G. Kotti M. Wiik A. V. Collins R. Brevadt M. J. Strachan R. K. (2016). Gait comparison of unicompartmental and total knee arthroplasties with healthy controls. Bone Jt. J. 98-B, 16–21. 10.1302/0301-620X.98B10.BJJ.2016.0473.R1 PMC5047137 27694511

[B32] Jordan S. S. DeFrate L. E. Kyung W. N. Papannagari R. Gill T. J. Li G. (2007). The *in vivo* kinematics of the anteromedial and posterolateral bundles of the anterior cruciate ligament during weightbearing knee flexion. Am. J. Sports Med. 35, 547–554. 10.1177/0363546506295941 17261571

[B33] Kutzner I. Trepczynski A. Heller M. O. Bergmann G. (2013). Knee adduction moment and medial contact force-facts about their correlation during gait. PLoS One 8, e81036. 10.1371/journal.pone.0081036 24312522 PMC3847086

[B34] Liddle A. D. Judge A. Pandit H. Murray D. W. (2014). Adverse outcomes after total and unicompartmental knee replacement in 101330 matched patients: a study of data from the National Joint Registry for England and Wales. Lancet 384, 1437–1445. 10.1016/S0140-6736(14)60419-0 25012116

[B35] Mathai B. Dhara S. Gupta S. (2022). Bone remodelling in implanted proximal femur using topology optimization and parameterized cellular model. J. Mech. Behav. Biomed. Mat. 125, 104903. 10.1016/j.jmbbm.2021.104903 34717117

[B36] Meireles S. Completo A. António Simões J. Flores P. (2010). Strain shielding in distal femur after patellofemoral arthroplasty under different activity conditions. J. Biomech. 43, 477–484. 10.1016/j.jbiomech.2009.09.048 20004900

[B37] Munford M. J. Stoddart J. C. Liddle A. D. Cobb J. P. Jeffers J. R. T. (2022). Total and partial knee arthroplasty implants that maintain native load transfer in the tibia. Bone Jt. Res. 11, 91–101. 10.1302/2046-3758.112.BJR-2021-0304.R1 PMC8882327 35168367

[B38] Ong K. L. Day J. S. Kurtz S. M. Field R. E. Manley M. T. (2009). Role of surgical position on interface stress and initial bone remodeling stimulus around hip resurfacing arthroplasty. J. Arthroplasty 24, 1137–1142. 10.1016/j.arth.2008.08.005 18823747

[B39] Pal B. Gupta S. New A. M. R. Browne M. (2010). Strain and micromotion in intact and resurfaced composite femurs: experimental and numerical investigations. J. Biomech. 43, 1923–1930. 10.1016/j.jbiomech.2010.03.019 20392448

[B40] Papannagari R. DeFrate L. E. Nha K. W. Moses J. M. Moussa M. Gill T. J. (2007). Function of posterior cruciate ligament bundles during *in vivo* knee flexion. Am. J. Sports Med. 35, 1507–1512. 10.1177/0363546507300061 17376856

[B41] Parvizi J. Jain N. Schmidt A. H. (2008). Periprosthetic knee fractures. J. Orthop. Trauma 22, 663–671. 10.1097/BOT.0b013e31816ed989 18827599

[B42] Pegg E. C. Walter J. D’Lima D. D. Fregly B. J. Gill H. S. Murray D. W. (2020). Minimising tibial fracture after unicompartmental knee replacement: a probabilistic finite element study. Clin. Biomech. 73, 46–54. 10.1016/j.clinbiomech.2019.12.014 PMC10135372 31935599

[B43] Pérez M. A. Fornells P. Doblaré M. García-Aznar J. M. (2010). Comparative analysis of bone remodelling models with respect to computerised tomography-based finite element models of bone. Comput. Methods Biomech. Biomed. Engin. 13, 71–80. 10.1080/10255840903045029 19697182

[B44] Petersen M. M. Olsen C. Lauritzen J. B. Lund B. (1995). Changes in bone mineral density of the distal femur following uncemented total knee arthroplasty. J. Arthroplasty 10, 7–11. 10.1016/S0883-5403(05)80094-4 7730833

[B45] Phillips A. T. M. Villette C. C. Modenese L. (2015). Femoral bone mesoscale structural architecture prediction using musculoskeletal and finite element modelling. Int. Biomech. 2, 43–61. 10.1080/23335432.2015.1017609

[B46] Prince J. M. Bernatz J. T. Binkley N. Abdel M. P. Anderson P. A. (2019). Changes in femoral bone mineral density after total knee arthroplasty: a systematic review and meta-analysis. Arch. Osteoporos. 14, 23. 10.1007/s11657-019-0572-7 30798359

[B47] Quilez M. P. Seral B. Pérez M. A. (2017). Biomechanical evaluation of tibial bone adaptation after revision total knee arthroplasty: a comparison of different implant systems. PLoS One 12, e0184361. 10.1371/journal.pone.0184361 28886100 PMC5590921

[B48] Rasnick R. Standifird T. Reinbolt J. A. Cates H. E. Zhang S. (2016). Knee joint loads and surrounding muscle forces during stair ascent in patients with total knee replacement. PLoS One 11, e0156282. 10.1371/journal.pone.0156282 27258086 PMC4892639

[B49] Safiri S. Kolahi A.-A. Smith E. Hill C. Bettampadi D. Mansournia M. A. (2020). Global, regional and national burden of osteoarthritis 1990-2017: a systematic analysis of the Global Burden of Disease Study 2017. Ann. Rheum. Dis. 79, 819–828. 10.1136/annrheumdis-2019-216515 32398285

[B50] Sawatari T. Tsumura H. Iesaka K. Furushiro Y. Torisu T. (2005). Three-dimensional finite element analysis of unicompartmental knee arthroplasty–the influence of tibial component inclination. J. Orthop. Res. 23, 549–554. 10.1016/j.orthres.2004.06.007 15885474

[B51] Scheiner S. Pivonka P. Hellmich C. (2016). Poromicromechanics reveals that physiological bone strains induce osteocyte-stimulating lacunar pressure. Biomech. Model. Mechanobiol. 15, 9–28. 10.1007/s10237-015-0704-y 26220453 PMC4779462

[B52] Scott C. E. H. MacDonald D. J. Howie C. R. (2019). ‘Worse than death’ and waiting for a joint arthroplasty. Bone Jt. J. 101, 941–950. 10.1302/0301-620X.101B8.BJJ-2019-0116.R1 PMC6681678 31362549

[B53] Soininvaara T. A. Harju K. A. L. Miettinen H. J. A. Kröger H. P. J. (2013). Periprosthetic bone mineral density changes after unicondylar knee arthroplasty. Knee 20, 120–127. 10.1016/j.knee.2012.10.004 23154036

[B54] Stoddart J. C. Dandridge O. Garner A. Cobb J. van Arkel R. J. (2021). The compartmental distribution of knee osteoarthritis – a systematic review and meta-analysis. Osteoarthr. Cartil. 29, 445–455. 10.1016/j.joca.2020.10.011 33253887

[B55] Stoddart J. C. Garner A. Tuncer M. Cobb J. P. van Arkel R. J. (2022). The risk of tibial eminence avulsion fracture with bi-unicondylar knee arthroplasty: a finite element analysis. Bone Jt. Res. 11, 575–584. 10.1302/2046-3758.118.BJR-2021-0533.R1 PMC9396920 35920206

[B56] Thoreau L. Morcillo Marfil D. Thienpont E. (2022). Periprosthetic fractures after medial unicompartmental knee arthroplasty: a narrative review. Arch. Orthop. Trauma Surg. 142, 2039–2048. 10.1007/s00402-021-04063-z 34268614

[B57] Tuncer M. Cobb J. P. Hansen U. N. Amis A. A. (2013). Validation of multiple subject-specific finite element models of unicompartmental knee replacement. Med. Eng. Phys. 35, 1457–1464. 10.1016/j.medengphy.2013.03.020 23647863

[B58] Turner A. W. L. Gillies R. M. Sekel R. Morris P. Bruce W. Walsh W. R. (2005). Computational bone remodelling simulations and comparisons with DEXA results. J. Orthop. Res. 23, 705–712. 10.1016/j.orthres.2005.02.002 16022980

[B59] Van Arkel R. J. Modenese L. Phillips A. T. M. Jeffers J. R. T. (2013). Hip abduction can prevent posterior edge loading of hip replacements. J. Orthop. Res. 31, 1172–1179. 10.1002/jor.22364 23575923 PMC3736148

[B60] van Jonbergen H. P. W. Innocenti B. Gervasi G. L. Labey L. Verdonschot N. (2012). Differences in the stress distribution in the distal femur between patellofemoral joint replacement and total knee replacement: a finite element study. J. Orthop. Surg. Res. 7, 28. 10.1186/1749-799X-7-28 22704638 PMC3471009

[B61] Van Jonbergen H. P. W. Koster K. Labey L. Innocenti B. Van Kampen A. (2010). Distal femoral bone mineral density decreases following patellofemoral arthroplasty: 1-year follow-up study of 14 patients. BMC Musculoskelet. Disord. 11, 74. 10.1186/1471-2474-11-74 20406477 PMC2864205

[B67] van Lenthe G. H. de Waal Malefijt M. C. Huiskes R. (1997). Stress shielding after total knee replacement may cause bone resorption in the distal femur. J. Bone Jt. Surg. 79 (1), 117–122. 10.1302/0301-620X.79B1.6808 9020459

[B62] Wada K. Price A. Gromov K. Lustig S. Troelsen A. (2020). Clinical outcome of bi-unicompartmental knee arthroplasty for both medial and lateral femorotibial arthritis: a systematic review—is there proof of concept? Arch. Orthop. Trauma Surg. 140, 1503–1513. 10.1007/s00402-020-03492-6 32529388

[B63] Wang H. Foster J. Franksen N. Estes J. Rolston L. (2018). Gait analysis of patients with an off-the-shelf total knee replacement versus customized bi-compartmental knee replacement. Int. Orthop. 42, 805–810. 10.1007/s00264-017-3622-z 28868567

[B64] Wiik A. V. Manning V. Strachan R. K. Amis A. A. Cobb J. P. (2013). Unicompartmental knee arthroplasty enables near normal gait at higher speeds, unlike total knee arthroplasty. J. Arthroplasty 28, 176–178. 10.1016/j.arth.2013.07.036 24099573 PMC3809509

[B65] Yoon C. Chang M. J. Chang C. B. Song M. K. Shin J. H. Kang S.-B. (2018). Medial tibial periprosthetic bone resorption and its effect on clinical outcomes after total knee arthroplasty: cobalt-chromium vs titanium implants. J. Arthroplasty 33, 2835–2842. 10.1016/J.ARTH.2018.04.025 29773278

[B66] Zhang M. Gregory T. Hansen U. Cheng C. K. (2020). Effect of stress-shielding-induced bone resorption on glenoid loosening in reverse total shoulder arthroplasty. J. Orthop. Res. 38, 1566–1574. 10.1002/jor.24711 32374418

