# Supplementary material for: Load transfer in bone after partial, multi-compartmental, and total knee arthroplasty
Source: Front Bioeng Biotechnol. 2024 Mar 8;12:1274496. doi: 10.3389/fbioe.2024.1274496 (PMC10957574; doi:10.3389/fbioe.2024.1274496)
Supplement: Supplementary file 1 [file DataSheet1.docx]

Supplementary Material

Load transfer in bone after Partial, Combined Partial and Total Knee Arthroplasty

Jennifer C Stoddart^1^, Amy Garner^2,3,4^, Mahmut Tuncer^5^, Andrew Amis^1^, Justin Cobb^2^, Richard van Arkel^1*^

*** Correspondence:** Richard van Arkel: r.vanarkel@imperial.ac.uk

# Subject-specific model characteristics

**Supplementary Table 1:** *Characteristics of the subject-specific finite element models*

|  | Age (years) | Height (cm) | Weight (kg) | Mean bone density (gcm^-3^) | Bone volume (cm^3^) |
| --- | --- | --- | --- | --- | --- |
| Male | 65 | 183 | 64 | 0.344 | 199 |
| Female | 81 | 152 | 91 | 0.296 | 154 |

**Supplementary Table 2:** *Implant sizes used for each of the subject-specific finite element models.*

|  | Tibia | | | Femur | | | |
| --- | --- | --- | --- | --- | --- | --- | --- |
|  | *UKA-M* | *UKA-L* | *TKA* | *UKA-M* | *UKA-L* | *PFA* | *TKA* |
| Male | C | E | 6 | Medium | Large | 4 | F |
| Female | B | D | 5 | Small | Medium | 3 | E |

**Supplementary Table 3:** *Material properties applied to each aspect of the finite element model*s (Tuncer et al., 2013).

| Material | Density ρ (gcm^-3^) | Elastic Modulus, E (MPa) | | | Poisson’s ratio |
| --- | --- | --- | --- | --- | --- |
| Proximal tibial cancellous bone | $\rho=0.04+0.00092 \text{HU}$ | | $E =\left\{ \begin{aligned} 311.1 \\ 26480\rho^{1.93} \\ 5230\rho^{2.39}+1000 \end{aligned} \right.\begin{matrix} \rho<0.1 \\ 0.1 <\rho<0.37 \\ \rho>1.5 \end{matrix}$ | 0.3 | |
| Distal femoral cancellous bone | $\rho=0.04+0.00092 \text{HU}$ | | $E =\left\{ \begin{aligned} 878.7 \\ 7850\rho^{1.49} \\ 4830\rho^{2.39} \end{aligned}\begin{matrix} \rho<0.23 \\ 0.23 <\rho<0.7 \\ \rho>1.5 \end{matrix} \right.$ | 0.3 | |
| Cortical bone | N/A | | 18,000 male/ 16,000 female | 0.33 | |
| CoCr implant | N/A | | 210,000 | 0.33 | |
| PE bearing | N/A | | 600 | 0.3 | |
| PMMA cement | N/A | | 1800 | 0.33 | |

# Load case boundary conditions

## Toe-off, gait

**Supplementary Table 4:** *Summary of the forces applied to the tibia in the toe-off, gait load case. Force direction unit vectors are given in terms of the tibial coordinate system. Force magnitudes are scaled to 91 kg bodyweight of the female model.*

| Tibia, Gait, 15^o^ flexion | | | | | |
| --- | --- | --- | --- | --- | --- |
| Loads |  | Magnitude (N) | Direction | | |
|  |  |  | x | y | z |
| Contact forces | Medial TF | 2357 | -0.01 | -0.08 | -0.99 |
|  | Lateral TF | 1022 | -0.01 | -0.08 | -0.99 |
| Muscle forces | Biceps Femoris (long head) | 203 | -0.16 | -0.26 | 0.95 |
|  | Biceps Femoris (short head) | 203 | -0.05 | -0.09 | 0.99 |
|  | Patellar tendon (quads) | 723 | -0.01 | 0.31 | 0.95 |
| Ligament forces | ACL (AM bundle) | 242 | 0.17 | -0.63 | 0.76 |
|  | ACL (PL bundle) | 109 | 0.18 | -0.65 | 0.74 |
|  | PCL | 149 | -0.30 | 0.57 | 0.77 |
|  | MCL | 138 | 0 | -0.21 | -0.98 |

**Supplementary Table 5:** *Summary of the forces applied to the femur in the toe-off, gait load case. Force direction unit vectors are given in terms of the femoral coordinate system. Force magnitudes are scaled to 91 kg bodyweight of the female model.*

| Femur, Gait, 15^o^ flexion | | | | | |
| --- | --- | --- | --- | --- | --- |
| Loads |  | Magnitude (N) | Direction | | |
|  |  |  | x | y | z |
| Contact forces | Medial TF | 2352 | 0.01 | 0.33 | 0.94 |
|  | Lateral TF | 1019 | 0.01 | 0.33 | 0.94 |
|  | Patellofemoral | 372 | 0.12 | -0.76 | 0.64 |
| Muscle forces | Gastrocnemius (medial) | 612 | 0.05 | -0.74 | -0.67 |
|  | Gastrocnemius (lateral) | 612 | -0.06 | -0.76 | -0.65 |
| Ligament forces | ACL | 351 | -0.17 | 0.41 | -0.90 |
|  | PCL | 149 | 0.30 | -0.75 | -0.59 |
|  | MCL | 138 | 0 | -0.05 | -0.99 |

## Weight-acceptance, Stair Ascent

**Supplementary Table 6:** Summary of the forces applied to the tibia in the weight-acceptance, stair ascent load case. Force direction unit vectors are given in terms of the tibial coordinate system. Force magnitudes are scaled to 91 kg bodyweight of the female model.

| *Tibia, Stair Ascent, 50^o^ flexion* | | | | | |
| --- | --- | --- | --- | --- | --- |
| *Loads* |  | Magnitude (N) | Direction | | |
|  |  |  | x | y | z |
| *Contact forces* | Medial TF | 2760 | 0.07 | -0.21 | -0.98 |
|  | Lateral TF | 1412 | 0.07 | -0.21 | -0.98 |
| *Muscle forces* | Biceps Femoris (long head) | 286 | -0.26 | -0.76 | 0.60 |
|  | Biceps Femoris (short head) | 295 | -0.16 | -0.58 | 0.80 |
|  | Patellar tendon (quads) | 2358 | 0.07 | 0.16 | 0.98 |
|  | Sartorius | 27 | -0.15 | -0.19 | 0.97 |
|  | Semitendinosus | 9 | -0.20 | -0.74 | 0.65 |
|  | Semimembranosus | 446 | -0.14 | -0.77 | 0.63 |
| *Ligament forces* | ACL (AM bundle) | 155 | 0.17 | -0.76 | 0.63 |
|  | PCL | 345 | -0.23 | 0.55 | 0.80 |

# Supplementary Table 7: Summary of the forces applied to the femur in the weight-acceptance, stair ascent load case. Force direction unit vectors are given in terms of the femoral coordinate system. Force magnitudes are scaled to 91 kg bodyweight of the female model.

| *Femur, Stair Ascent, 50^o^ flexion* | | | | | |
| --- | --- | --- | --- | --- | --- |
| *Loads* |  | Magnitude (N) | Direction | | |
|  |  |  | x | y | z |
| *Contact forces* | Medial TF | 2766 | -0.07 | 0.89 | 0.46 |
|  | Lateral TF | 1415 | -0.07 | 0.89 | 0.46 |
|  | Patellofemoral | 2358 | 0.15 | -0.92 | 0.36 |
| *Muscle forces* | Gastrocnemius (medial) | 652 | 0.07 | -0.97 | -0.23 |
|  | Gastrocnemius (lateral) | 196 | -0.04 | -0.97 | -0.26 |
| *Ligament forces* | ACL | 155 | -0.17 | 0.01 | -0.99 |
|  | PCL | 345 | 0.23 | -0.97 | -0.09 |

## Maximal loading, Sit-to-Stand

# Supplementary Table 8: Summary of the forces applied to the tibia in the point of maximal loading in the sit-to-stand load case. Force direction unit vectors are given in terms of the tibial coordinate system. Force magnitudes are scaled to 91 kg bodyweight of the female model.

| *Tibia, Sit-to-Stand, 90^o^ flexion* | | | | | |
| --- | --- | --- | --- | --- | --- |
| *Loads* |  | Magnitude (N) | Direction | | |
|  |  |  | x | y | z |
| *Contact forces* | Medial TF | 1591 | 0.04 | -0.17 | -0.99 |
|  | Lateral TF | 2239 | 0.04 | -0.17 | -0.99 |
| *Muscle forces* | Biceps Femoris (long head) | 268 | -0.31 | -0.95 | -0.05 |
|  | Biceps Femoris (short head) | 268 | -0.24 | -0.95 | 0.20 |
|  | Patellar tendon (quads) | 1921 | 0.08 | 0.02 | 0.99 |
| *Ligament forces* | ACL | 0 | - | - | - |
|  | PCL | 493 | -0.20 | 0.39 | 0.90 |
|  | MCL | 380 | 0 | -0.30 | 0.95 |

**Supplementary Table 9:** Summary of the forces applied to the femur in the point of maximal loading in the sit-to**-**stand load case. Force direction unit vectors are given in terms of the femoral coordinate system. Force magnitudes are scaled to 91 kg bodyweight of the female model.

| *Femur, Sit-to-Stand, 90^o^ flexion* | | | | | |
| --- | --- | --- | --- | --- | --- |
| *Loads* |  | Magnitude (N) | Direction | | |
|  |  |  | x | y | z |
| *Contact forces* | Medial TF | 1591 | -0.04 | 0.99 | -0.17 |
|  | Lateral TF | 2239 | -0.04 | 0.99 | -0.17 |
|  | Patellofemoral | 2648 | 0.48 | -0.34 | 0.81 |
| *Muscle forces* | Gastrocnemius (medial) | 133 | 0.07 | -0.92 | 0.39 |
|  | Gastrocnemius (lateral) | 133 | -0.04 | -0.90 | 0.44 |
| *Ligament forces* | ACL | 0 | - | - | - |
|  | PCL | 493 | 0.20 | -0.90 | 0.39 |
|  | MCL | 380 | 0 | -0.95 | -0.30 |

# Summary statistics for toe-off gait load case

**Supplementary Table 10:** *Summary statistics describing the overstraining and strain shielding distribution during toe-off in gait, throughout the distal femur and proximal tibia of the female model*

| Arthroplasty | | Mean (%) | Variance | Skew |
| --- | --- | --- | --- | --- |
| *Distal Femur* | UKA-M | -3.5 | 223.8 | -2.2 |
|  | UKA-L | -4.5 | 208.5 | -3.0 |
|  | PFA | -7.3 | 204.1 | -2.8 |
|  | BCA-M | -8.1 | 439.6 | -1.1 |
|  | BCA-L | -7.8 | 374.3 | -1.7 |
|  | Bi-UKA | -5.2 | 488.8 | -0.9 |
|  | TCA | -7.8 | 1501.5 | 10.2 |
|  | TKA | -30.2 | 797.6 | 0.3 |
| *Proximal Tibia* | UKA-M | 0.4 | 1017.7 | 2.7 |
|  | UKA-L | 6.4 | 1664.7 | 6.1 |
|  | Bi-UKA | 2.4 | 1646.1 | 3.2 |
|  | TKA | 21.3 | 10064 | 2.4 |

# Additional load case results

## Stair ascent


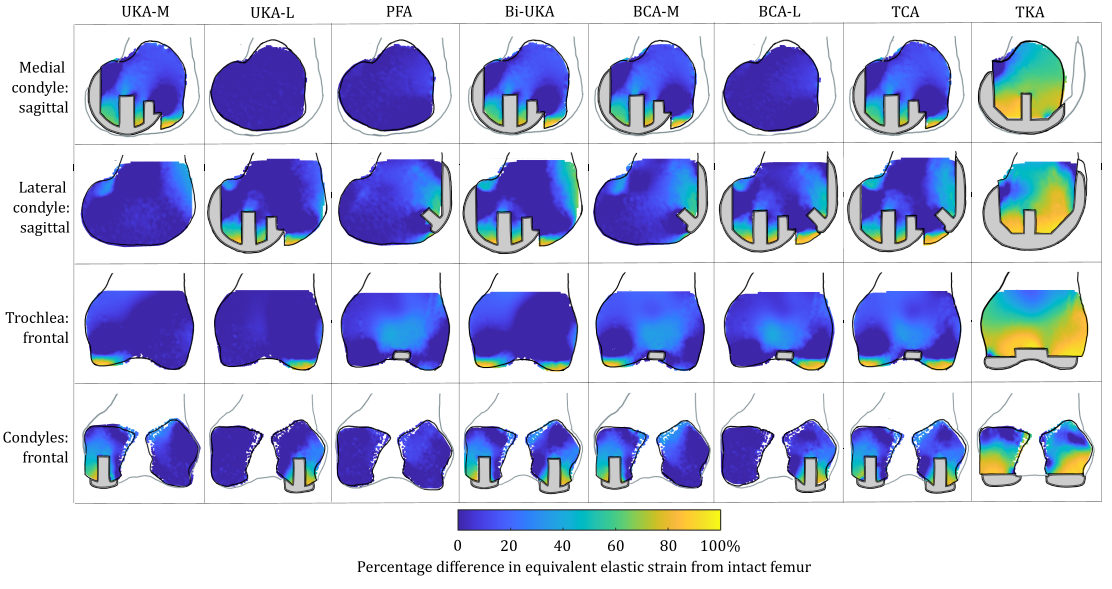


**Supplementary Figure 1**: Contour plots showing the degree of strain shielding predicted during weight-acceptance in stair ascent, in the implanted femur compared to the intact femur.


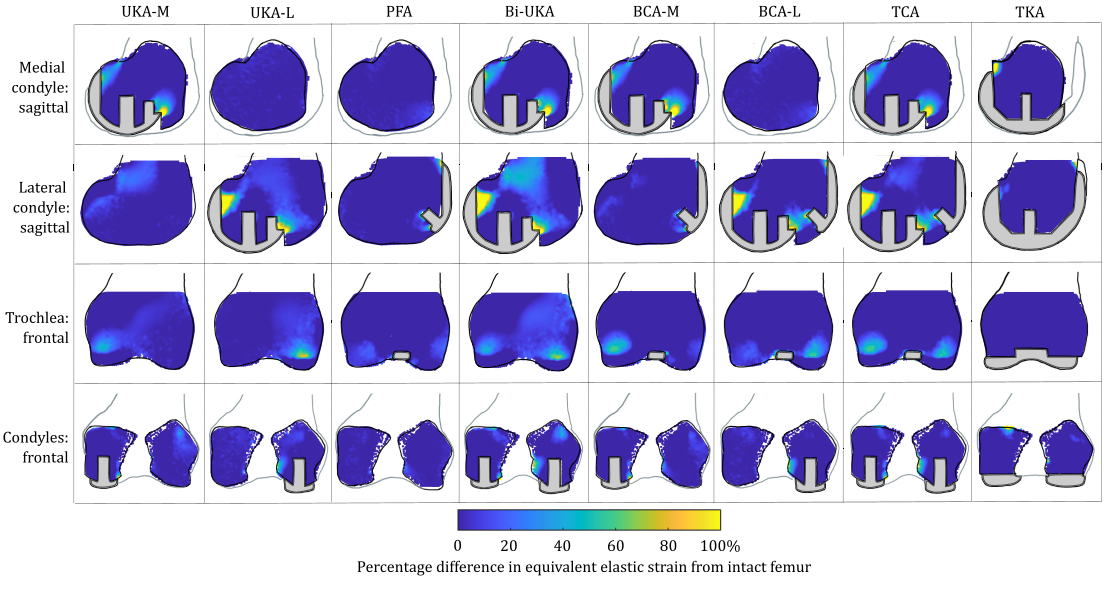


**Supplementary Figure 2:** Contour plots showing the degree of overstraining predicted during weight-acceptance in stair ascent, in the implanted femur compared to the intact femur.


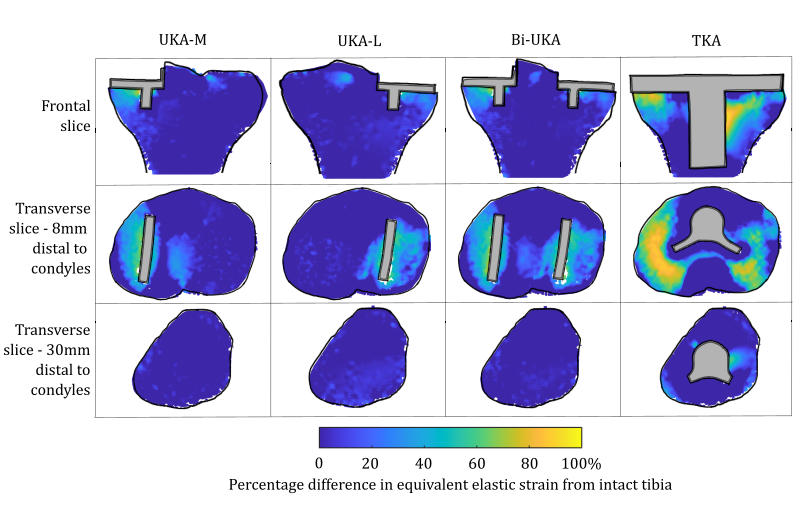


**Supplementary Figure 3:** Contour plots showing the degree of strain shielding predicted during weight-acceptance in stair ascent, in the implanted tibia compared to the intact tibia.


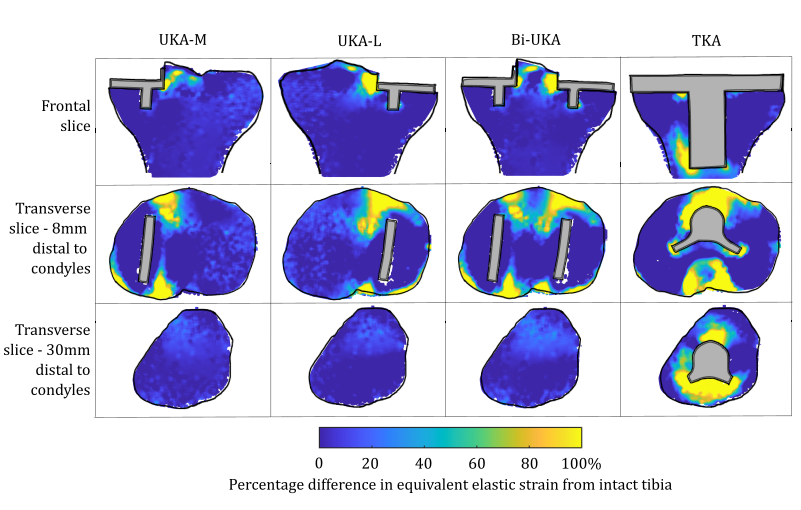


**Supplementary Figure 4:** Contour plots showing the degree of overstraining predicted during weight-acceptance in stair ascent, in the implanted tibia compared to the intact tibia.


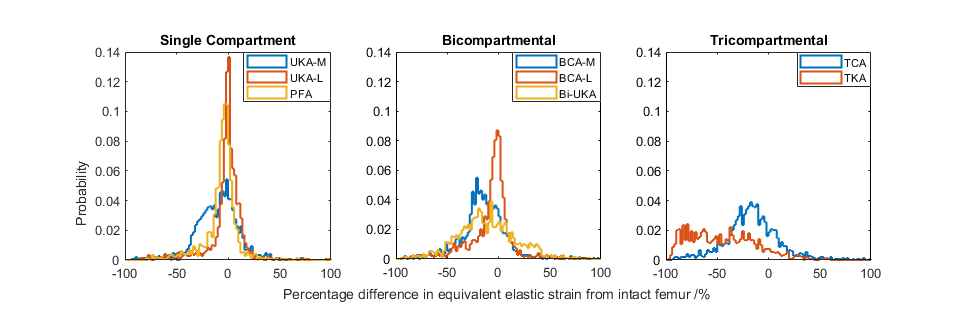


**Supplementary Figure 5:** Histograms showing the distribution of strain shielding (negative x-axis) and overstraining (positive x-axis) throughout the volume of the implanted distal femur during weight-acceptance in stair ascent.


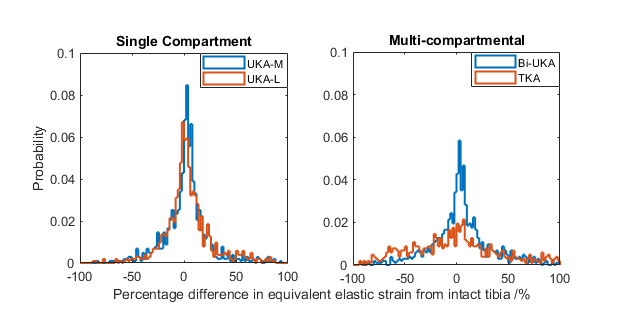


**Supplementary Figure 6:** Histograms showing the distribution of strain shielding (negative x-axis) and overstraining (positive x-axis) throughout the volume of the implanted proximal tibia during weight-acceptance in stair ascent.

## Sit-to-stand


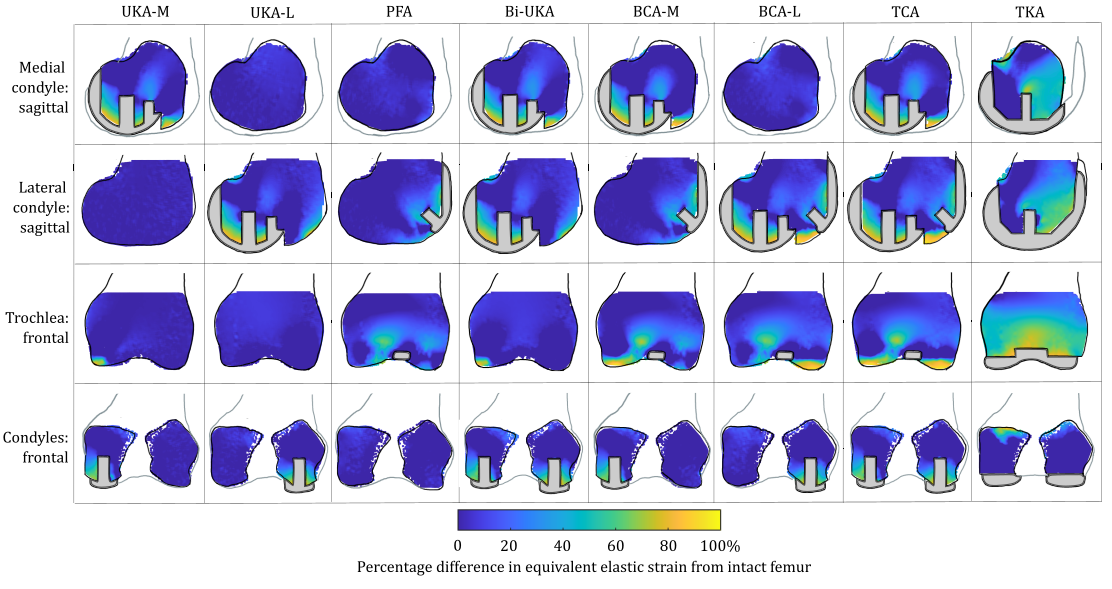


**Supplementary Figure 7:** Contour plots showing the degree of strain shielding predicted during sit-to-stand, in the implanted femur compared to the intact femur.


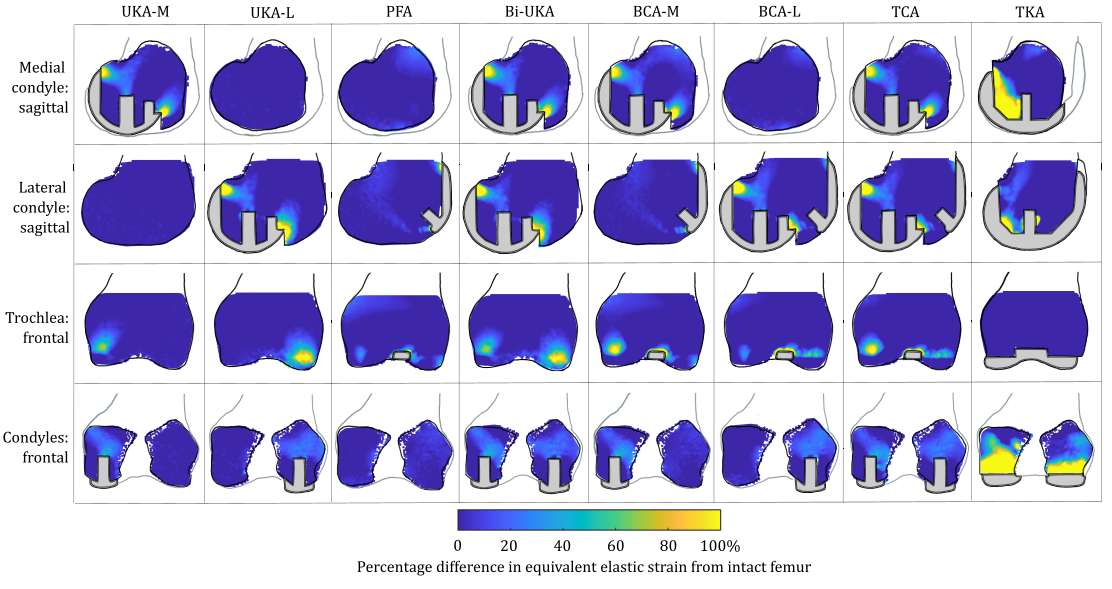


**Supplementary Figure 8:** Contour plots showing the degree of overstraining predicted during sit-to-stand, in the implanted femur compared to the intact femur.


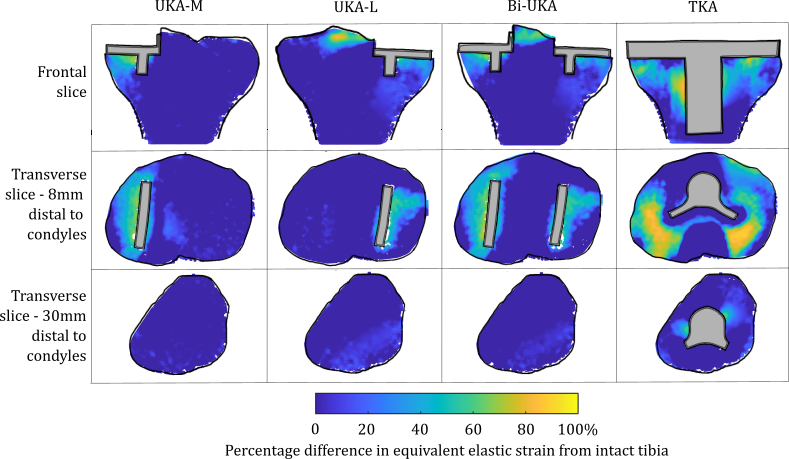


**Supplementary Figure 9:** Contour plots showing the degree of strain shielding predicted during sit-to-stand, in the implanted tibia compared to the intact tibia.


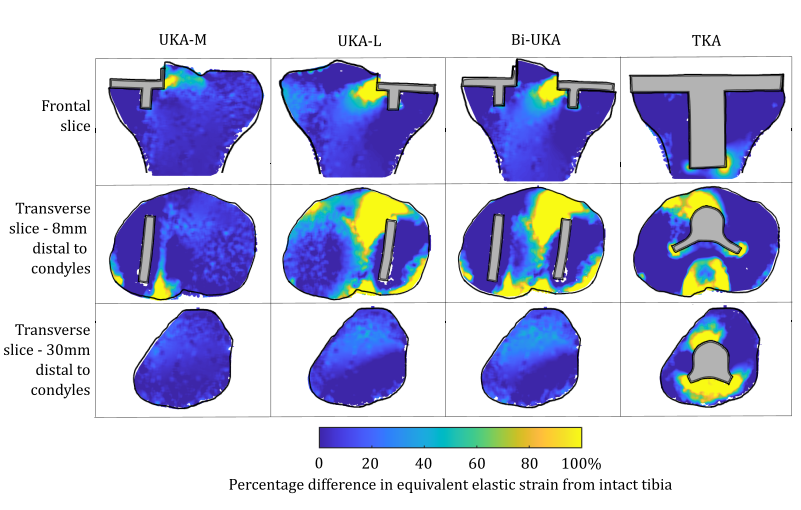


**Supplementary Figure 10:** Contour plots showing the degree of overstraining predicted during sit-to-stand, in the implanted tibia compared to the intact tibia.


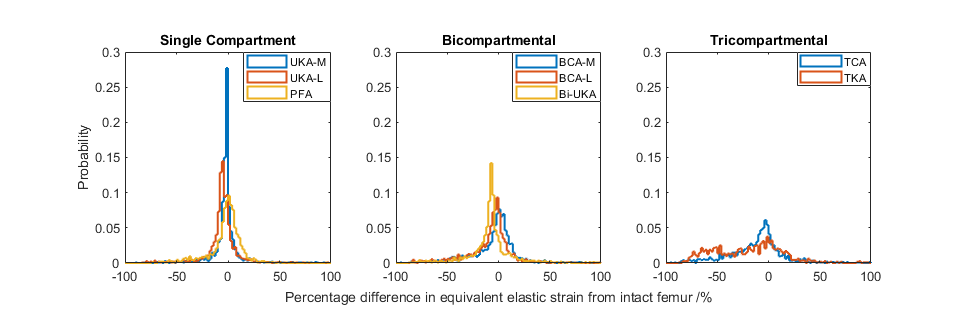


**Supplementary Figure 11:** Histograms showing the distribution of strain shielding (negative x-axis) and overstraining (positive x-axis) throughout the volume of the implanted distal femur during sit-to-stand.


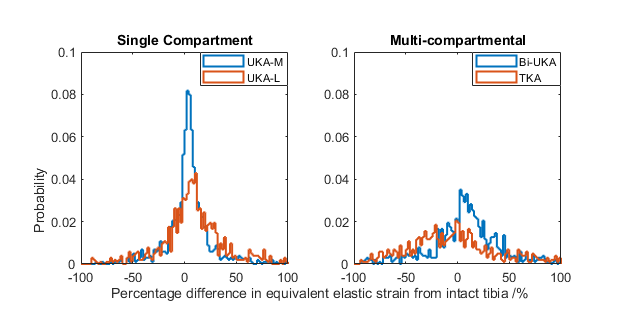


**Supplementary Figure 12:** Histograms showing the distribution of strain shielding (negative x-axis) and overstraining (positive x-axis) throughout the volume of the implanted proximal tibia during sit-to-stand.

# Bone volume removed for implantation

The amount of bone removed was roughly equal for each of the PKAs (Supplementary Figure 13). As the individual implants were not moved when used in combination, the volume of bone removed for CPKA involving two compartments doubled, and tripled for TCA. Conversely, the amount of bone removed in the femur after TKA was >9 times greater than for PKA. Compared to TCA, it was 3 times greater. This disparity was less severe in the tibia, with the bone removed in TKA 4 times greater than in UKA-M and double that removed for Bi-UKA.


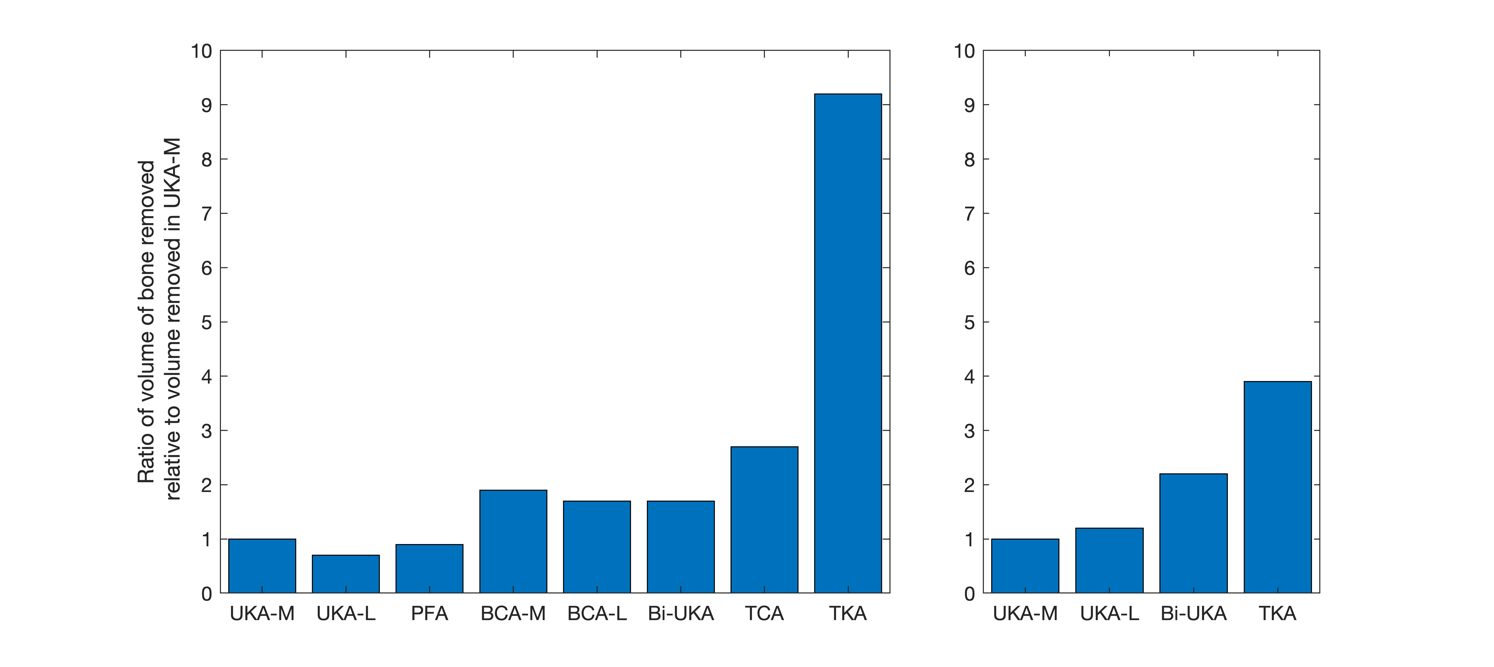


**Supplementary Figure 13:** Bar chart indicating the ratio of the volume of bone removed in each arthroplasty procedure for the female model in the femur (left) and tibia (right), when compared to the volume of bone removed in UKA-M in each bone respectively.
